# Patterns of asexual reproduction of the soybean aphid, *Aphis glycines* (Matsumura), with and without the secondary symbionts *Wolbachia* and *Arsenophonus,* on susceptible and resistant soybean genotypes

**DOI:** 10.3389/fmicb.2023.1209595

**Published:** 2023-08-31

**Authors:** Rosanna Giordano, Everett P. Weber, Ryan Mitacek, Alejandra Flores, Alonso Ledesma, Arun K. De, Theresa K. Herman, Felipe N. Soto-Adames, Minh Q. Nguyen, Curtis B. Hill, Glen L. Hartman

**Affiliations:** ^1^Institute of Environment, Florida International University, Miami, FL, United States; ^2^Puerto Rico Science Technology and Research Trust, San Juan, Puerto Rico; ^3^Office of Institutional Research, Dartmouth College, Hanover, NH, United States; ^4^Department of Crop Sciences, University of Illinois Urbana-Champaign, Urbana, IL, United States; ^5^Department of Physiology and Biophysics, School of Medicine, Case Western Reserve University, Cleveland, OH, United States; ^6^College of Agricultural, Consumer and Environmental Sciences, University of Illinois Urbana-Champaign, Urbana, IL, United States; ^7^Animal Sciences Division, ICAR-Central Island Agricultural Research Institute, Port Blair, India; ^8^USDA-Agricultural Research Service, Urbana, IL, United States; ^9^Division of Plant Industry, Florida Department of Agriculture and Consumer Services, Gainesville, FL, United States; ^10^Neochromosome, Inc., Long Island City, NY, United States; ^11^Agricen Sciences, Pilot Point, TX, United States

**Keywords:** soybean aphid, reproduction, resistant soybean varieties, symbionts, *Wolbachia*, *Arsenophonus*, *Hamiltonella*

## Abstract

Plant breeding is used to develop crops with host resistance to aphids, however, virulent biotypes often develop that overcome host resistance genes. We tested whether the symbionts, *Arsenophonus* (*A*) and *Wolbachia* (*W*), affect virulence and fecundity in soybean aphid biotypes Bt1 and Bt3 cultured on whole plants and detached leaves of three resistant, *Rag1*, *Rag2* and *Rag1 + 2,* and one susceptible, *W82*, soybean genotypes. Whole plants and individual aphid experiments of *A. glycines* with and without *Arsenophonus* and *Wolbachia* did not show differences in overall fecundity. Differences were observed in peak fecundity, first day of deposition, and day of maximum nymph deposition of individual aphids on detached leaves. Bt3 had higher fecundity than Bt1 on detached leaves of all plant genotypes regardless of bacterial profile. Symbionts did not affect peak fecundity of Bt1 but increased it in Bt3 (*A*+*W*+) and all Bt3 strains began to deposit nymphs earlier than the Bt1 (*A*+*W*−). *Arsenophonus* in Bt1 delayed the first day of nymph deposition in comparison to aposymbiotic Bt1 except when reared on *Rag1 + 2*. For the Bt1 and Bt3 strains, symbionts did not result in a significant difference in the day they deposited the maximum number of nymphs nor was there a difference in survival or variability in number of nymphs deposited. Variability of number of aphids deposited was higher in aphids feeding on resistant plant genotypes. The impact of *Arsenophonus* on soybean aphid patterns of fecundity was dependent on the aphid biotype and plant genotype. *Wolbachia* alone had no detectable impact but may have contributed to the increased fecundity of Bt3 (*A*+*W*+). An individual based model, using data from the detached leaves experiment and with intraspecific competition removed, found patterns similar to those observed in the greenhouse and growth chamber experiments including a significant interaction between soybean genotype and aphid strain. Combining individual data with the individual based model of population growth isolated the impact of fecundity and host resistance from intraspecific competition and host health. Changes to patterns of fecundity, influenced by the composition and concentration of symbionts*,* may contribute to competitive interactions among aphid genotypes and influence selection on virulent aphid populations.

## Introduction

Aphids feed on plant phloem, a source of food composed mostly of diluted sugars, amino acids, and a range of proteins and RNA molecules, some produced in response to environmental stresses (van Bel and Gaupels, 2004; Lough and Lucas, 2006; Turgeon and Wolf, 2009). As with other insects that feed on nutrient-poor food, aphids are associated with intracellular symbiotic organisms that contribute to their acquisition of nutrients. Most species within the Aphidoideae, except for members of the Cerataphidini tribe, who are colonized by symbiotic yeast (Fukatsu and Ishikawa, 1992), are associated with the obligate symbiotic bacteria *Buchnera aphidicola* (Buchner, 1965; Munson et al., 1991a,b; Vogel and Moran, 2013), a relationship estimated to have begun *circa* 150 Ma (Moran et al., 1993; Von Dohlen and Moran, 2000). This long association has rendered them inextricably tied; aphids deprived of *B. aphidicola* cannot reproduce and attempts at *in vitro* culturing of the bacterium have failed. The obligate *B. aphidicola* is not the only symbiont associated with aphids. Insects in general, and aphids in particular, are associated with a panoply of facultative or secondary symbionts, some of which have been shown to render fitness benefits to the host (Montllor et al., 2002; Tsuchida et al., 2002; Oliver et al., 2003, 2007; Tsuchida et al., 2004; Scarborough et al., 2005; Russell and Moran, 2006; Łukasik et al., 2012, 2013; Henry et al., 2013). The presence of symbiotic bacteria in insects may affect their interaction with plants including facilitating the colonization of resistant plants (Hebert et al., 2007; Francis et al., 2010; Frago et al., 2012; Biere and Tack, 2013). Moreover, the intimate association between aphids and plants has facilitated the transfer of bacteria across these two kingdoms, allowing the opportunity for bacteria to commonly infect aphids and establish new niches (Caspi-Fluger and Zchori-Fein, 2010; Li et al., 2017).

The soybean aphid, *Aphis glycines* Matsumara 1917 (Hemiptera, Aphididae) is native to Eastern Asia (Wang et al., 1994; Van Der Berg et al., 1997; Blackman and Eastop, 2000) and a recent invader in North America where, after its detection in 2000, it quickly spread throughout the midwestern U.S. and southern provinces of Canada (Hunt et al., 2003; Venette and Ragsdale, 2004; Giordano et al., 2020). The spread of the soybean aphid was facilitated by the diffused availability of the primary and overwintering host plant, invasive plant species *Rhamnus cathartica* L. (Buckthorn) (Ragsdale et al., 2004), and by widespread cultivation of soybean, *Glycine max,* the secondary and summer host, in the agricultural landscape of North America, where there is also a general lack of geographical impediments to aerial dispersal (Wallin and Loonan, 1971; Irwin et al., 1988; Loxdale et al., 1993; Irwin et al., 2007). As observed with other invasive species (Elton, 2000), the soybean aphid has reached higher population densities and confers greater damage to the soybean crop in its invasive range compared to its native range (Liu et al., 2004; Ragsdale et al., 2004; Wu et al., 2004; Tilmon et al., 2011). If left untreated, damage due to the soybean aphid can cause significant yield losses to the soybean crop (Song et al., 2006; Kim C.S. et al., 2008; Johnson et al., 2009; Song and Swinton, 2009; Ragsdale et al., 2011). Soybean pest control is largely managed with neonicotinoid pesticide sprays or seed treatments (Johnson et al., 2009; Ragsdale et al., 2011; Myers and Hill, 2014; Bahlai et al., 2015). However, the widespread use of neonicotinoids is likely to be curtailed as they are implicated in the decline of pollinators and other insects with a ripple effect on their invertebrate and vertebrate predators (Di Prisco et al., 2013; Hallmann et al., 2014; Sánchez-Bayo, 2014). Sustainable alternatives include biological control measures (McCarville and O’Neal, 2012; Hallett et al., 2013; Heimpel et al., 2013) and host plant resistance (Li et al., 2004; Hill et al., 2004a,b, 2006a,b, 2012; Mardorf et al., 2010; McCarville and O’Neal, 2012, 2013; Wiarda et al., 2012; Hesler et al., 2013; Fox et al., 2014; McCarville et al., 2014). However, before the widespread field use of soybean aphid resistant genotypes could be completed, four soybean aphid biotypes able to overcome the resistance were identified (Kim K.-S. et al., 2008; Hill et al., 2010, 2012; Alt and Ryan-Mahmutagic, 2013; Pawlowski et al., 2014).

Resistant plant genotypes are a valuable, effective, and ecologically sustainable tool not only to control damage by aphids but also to limit the spread of diseases that affect soybean (i.e., Neupane et al., 2019; Barros et al., 2023). However, 72 virulent aphid biotypes have developed among 17 aphid species affecting at least 10 crop plants, with the greatest number of biotypes seen in *Diuraphis noxia*, the Russian wheat aphid, on wheat (Smith and Chuang, 2014). The mechanisms of aphid virulence are not well understood. Evidence, however, suggests that anatomical structures and chemical responses associated with the intake and digestion of plant phloem, and interactions with symbionts may also play a role (Bansal et al., 2013; Smith and Chuang, 2014; Bansal and Michel, 2015).

The specific way virulent soybean aphid biotypes overcome resistance genes in soybean is not known (Natukunda and MacIntosh, 2020). Previous reports with other aphids suggest that the ability of aphids to overcome plant resistance may be the result of mutation, gene regulation, gene amplification (Bass and Field, 2011; Bass et al., 2013; Feyereisen et al., 2015) and/or contributions of bacterial symbionts (Zytynska and Weisser, 2016). The soybean aphid harbors *Buchnera*, as with the great majority of aphids, as well as *Arsenophonus*, *Wolbachia* and *Hamiltonella*. *Arsenophonus* (Wille and Hartman, 2009; Bansal et al., 2013; Wenger and Michel, 2013; Wulff et al., 2013), is a bacterium that infects a variety of arthropod hosts (Duron et al., 2008; Nováková et al., 2009; Jousselin et al., 2013). Host effects attributed to the action of *Arsenophonus* range from male-killing in parasitic wasps (Huger et al., 1985; Werren et al., 1986; Gherna et al., 1991; Darby et al., 2010; Duron et al., 2010) to protection from parasitism in psyllids (Hansen et al., 2007). *Arsenophonus* infections in Asian and US populations of the soybean aphid have been reported (Bansal et al., 2013; Wulff et al., 2013). Despite the widespread occurrence of *Arsenophonus*, studies have shown it did not confer protection to soybean aphids from attack by three parasitoid species or by the aphid fungal pathogen *Pandora neoaphidis* (Remaudiere & Hennebert) (Wulff et al., 2013). *Arsenophonus* infection also did not influence soybean aphid virulence on *Rag* soybean aphid resistant genotypes, although infected aphids developed higher populations than the corresponding uninfected isolines (Wulff and White, 2015).

The soybean aphid is infected with the widely occurring Rickettsial arthropod symbiont *Wolbachia,* as well as *Hamiltonella,* and several extracellular bacteria (Bai et al., 2010; Liu et al., 2012; Bansal et al., 2013). *Wolbachia* have thus far been reported solely from invertebrates where they can elicit a range of effects from cytoplasmic incompatibility to male-killing (Stevens et al., 2001; Fenn and Blaxter, 2007; Werren et al., 2008; Kaur et al., 2021). *Hamiltonella defensa* has been documented in aphids, psyllids and whiteflies (Clark et al., 1992; Sandström et al., 2001; Russell et al., 2013). In aphids it has been shown to provide protection against parasitoid wasps (van der Wilk et al., 1999; Oliver et al., 2003, 2005, 2007; Ferrari et al., 2004; Moran et al., 2005; Bensadia et al., 2006; Degnan and Moran, 2007).

The association of Rickettsia with plants is exceedingly rare and a single report exists of a plant-pathogenic Rickettsia, causing papaya bunchy top disease (Davis et al., 1998). Evidence thus far indicates that the plant environment is not favorable for *Wolbachia* reproduction (Perlman et al., 2006; Weinert et al., 2009). We could not identify any report of *H. defensa* infecting plants. Conversely, *Arsenophonus* includes two well-characterized species, *Phlomobacter fragariae* and *Arsenophonus phytopathogenics,* that have been reported to be restricted to the phloem of plants and dependent on inter-plant transmission by their planthopper host: *Cixius wagneri* (China) (Hemiptera: Cixiidae) and *Pentastiridius leporinus* (L.) (Hemiptera: Cixiidae) respectively (Bressan et al., 2009; Bressan, 2014).

Given the influence that both intra-and extra-cellular bacterial symbionts have been shown to exert on their hosts, whether invertebrate or vertebrate, understanding the impact of bacterial infection on the traits of a major agricultural pest such as the soybean aphid may lead to important insights regarding their role as pests. The rapid development of soybean aphid virulence on resistant genotypes is of special concern, as resistant soybean genotypes hold the promise of providing pest control while minimizing detrimental impacts to the environment caused by pesticides (Natukunda and MacIntosh, 2020). We therefore tested whether *Arsenophonus* and *Wolbachia* have an effect on the expression of virulence and fecundity in the soybean aphid using two well-established laboratory strains of *A. glycines* and their corresponding *Arsenophonus* and *Wolbachia* free equivalents reared on whole plants and detached leaves of resistant and susceptible soybean genotypes.

## Materials and methods

We conducted three no-choice fecundity experiments. Two experiments introduced a fixed number of aphids to a caged whole plant (greenhouse and growth chamber experiments). A third experiment consisted of individual aphids reared on single detached plant leaves in petri dishes to determine the fecundity of individual aphids (detached leaves experiment). Analysis of the detached leaves experiment included an individual based model simulating the whole plant experiments using the detached leaves data.

### Soybean genotypes and aphid strains

All experiments used four soybean plant genotypes obtained from Brian Diers at the University of Illinois. Three were soybean aphid-resistant lines: (1) LD11-4576a (*Rag1*), (2) LD11-5431a (*Rag2*), (3) LD10-30014 (*Rag1*, *Rag2*), and (4) Williams 82 (*W82*) a line that has no known resistance to soybean aphid feeding.

Five soybean aphid strains were used with varying symbiont profiles (Supplementary Table S1): (1) Bt1 (*A*+*W*−), avirulent on *Rag1*, *Rag2* and *Rag1 + 2* soybean plants (Li et al., 2004; Hill et al., 2004a,b, 2006a,b), was collected in Urbana, IL shortly after the soybean aphid was first detected in North America and kept in culture in our laboratory since 2000. The genome of this strain was recently sequenced (Giordano et al., 2020; Mathers, 2020; Wenger et al., 2020). All strains used harbor the obligate symbiont *Buchnera*. We determined that the Bt1 laboratory strain was infected with *Arsenophonus* and *Hamiltonella* but not infected with *Wolbachia* which it likely lost while in culture because all world-wide field populations tested have been found to be infected with *Wolbachia* (Giordano et al., in preparation). (2) Bt1 (*A*−*W*−), is an isofemale line derived from Bt1 via the microinjection of ampicillin (Sigma, St. Louis, MO). (3) Bt3 (*A*+*W*+), is avirulent on *Rag1* and virulent on *Rag2* soybean plants (Hill et al., 2010) and has been in culture since 2007 when it was collected from its overwintering host *Rhamnus frangula* in Springfield Fen, Indiana. This Bt3 laboratory strain is infected with *Hamiltonella*, *Arsenophonus* and *Wolbachia*. (4) Bt3 (*A*−*W*+) strain was derived from Bt3 via microinjection with ampicillin (Sigma, St. Louis, MO) while (5) Bt3 (*A*−*W*−) was derived by the microinjection of Bt3 with doxycycline hyclate (Sigma, St. Louis, MO). It was not possible to clear the strain of *Wolbachia* without also clearing *Arsenophonus.* We therefore could not generate a Bt3 (*A*+*W*−) strain. Results from the screening and curing of aphid strains can be found in Supplementary Figures S1–S3. Primers used for the screening can be found in Supplementary Tables S2, S3. Methods used to microinject aphids with antibiotics to eliminate specific bacteria can be found in Supplementary material.

**S**oybean aphid strains used in all experiments were cultured on detached leaves of soybean variety *W82* placed in petri dishes (100 × 20 mm) at 25°C under a 16 h photoperiod. Fifteen to 20 apterous adult females were placed on each leaf and allowed to lay nymphs. Twenty-two to three-day-old nymphs were transferred to fresh soybean leaves and reared to adulthood. Offspring that were 2–3 day old produced by this second generation of nymphs were utilized for the experiments.

### Plant cultivation, insect cages and greenhouse and growth chamber experiments

All plants were grown in 13 cm diameter plastic pots using soil-less medium (LC1 Sunshine Mix, Sungro Horticultural Distribution Inc., Agawam, MA) and 15 mL of slow-release fertilizer pellets (Osmocote 19-6-12) spread evenly over the growth medium to an approximate density of 2–3 pellets per cm^2^ following planting. Three seeds of each soybean genotype were planted per pot and thinned to one plant per pot after emergence, then grown to the VC stage for use in growth chamber or greenhouse experiments or V1–V3 stage for use in detached leaf experiments.

Plants for the growth chamber experiment were reared in a Conviron PGR15 (Manitoba, Canada) illuminated with 500 μmol m^2^s^−1^ PAR fluorescent and incandescent lamps programmed for a 16-h photoperiod at a constant 22°C. Plants remained in the same growth chamber under the same conditions after the application of aphids and containment cages.

Growth chamber experiment cages consisted of a clear flexible plastic tube 10.5 × 45 cm with an opaque plastic top and two opposing, rectangular side silk (Rose Brand, Sun Valley, CA; vanilla, non-flame retardant, SILK0031) panels of 6 × 25.5 cm placed 3 cm from the top for ventilation. Cages for the greenhouse experiment were 17.78 × 17.78 × 40.64 cm and consisted of a wood frame and bottom with a plexiglass top and paneled on all four sides with silk. For both experiments, single VC-age plants were placed inside each cage and inoculated with 20 soybean aphid nymphs. Aphid populations on whole plants were enumerated 14 days after inoculation. We used a nonparametric scale to rate the health of the plants based on that of Hill et al. (2006a): (1) Good—little to no evidence of damage; (2) Fair—some chlorosis; (3) Poor—chlorosis with some leaf damage; (4) Very Poor—chlorosis, leaf distortion and stunting.

### Detached leaf experiment

In the detached leaves experiment, fecundity of individual aphids was followed through their entire life cycle. For this experiment single unifoliate leaves with petioles were obtained from plants in the V1 or V2 stage of each variety and placed in individual petri dishes (100 × 20 mm) with a small cotton pad imbued with water wrapped around the petiole. A single aphid was placed on the top (adaxial) surface of each leaf with a fine sable paintbrush. Petri dishes were wrapped around the edge and sealed with parafilm (Bemis, Neenah, WI). Dishes were arranged on stainless steel trays so that leaves were fully illuminated and their order on the trays was rotated daily. To ensure that no contamination occurred between aphid strains in separate dishes, each strain was placed in a separate incubator. Trays were rotated daily in each chamber. Petri dishes were monitored daily, leaves were changed every 4 days, and deposited nymphs were counted and removed daily for 18 days. Petri dishes in which aphids trapped themselves in the cotton and died were eliminated from the study. The experiment was conducted at 25°C in Percival reach-in plant growth chambers, Model E-22 L, with a light intensity of 500 micromoles/m^2^/s, from sixteen 17 W cool white, fluorescent lamps and two 25 W incandescent lamps with a cycle of 16 h light and 8 h dark.

### Experimental design

The greenhouse and growth chamber experiments used three replicates of each of the 20 treatments (four soybean genotypes and five aphid strains) for a total of 60 plants per experiment. For the greenhouse experiment, caged pots were placed in pairs, in trays without holes and arranged using a randomized block design to account for the pairing. For the detached leaf experiment, all 20 treatments (four soybean genotypes and five aphid strains) were included. The experiment began with 12 replicates for each treatment, except for Bt3 (*A*−*W*−) reared on the susceptible *W82* soybean leaves that had 13 replicates. Two trials, run at different times, were conducted for the plant growth chamber experiment and a single trial was conducted in the greenhouse.

### PCR test for *Arsenophonus* and *Wolbachia* bacteria

The infection profile of test *A. glycines* laboratory strains, Bt1 (*A*+*W*−), and Bt3 (*A*+*W*+), was determined in the following manner: DNA was extracted from freshly killed aphids using the DNA Micro Kit (Qiagen, Valencia, CA) following the manufacturer’s protocol but with the following two changes: a 10 min incubation at 70°C after addition of the lysis buffer (AL) and the use of Wizard SV Mini columns (Promega) as these give a higher DNA yield. Specimens were macerated with a polypropylene pestle (Bel-Art Products) while viewing the specimen under a dissecting scope. Individual aphid specimens were tested with *Wolbachia* and *Arsenophonus* specific primers both as a screening tool during the process of generating the cured Bt1 and Bt3 lines as well as to confirm the infection profile of a subset of the initial and final aphids in the whole plant experiments and all the aphids that survived to the end of the detached leaves experiment. The bacterial screening primers and their respective protocols used were as follows: (1) *Wolbachia* screen: *dnaA* 2F (5′-acaattggttatatcagctg-3′) and *dnaA* 2R (5′-tacatagctatttgygttgg-3′) (Casiraghi et al., 2003) (95°C 3 min; followed by 35 cycles of 95°C 30s, 52°C 30s, 72°C 1 min); *Arsenophonus* screen: Gly1-2F (5′-cgcgtmaagccaatctaagattg-3′) designed for this work and 480R (“-cacggtactggttcactatcggtc-3′) (Sandström et al., 2001) (95°C 3 min, followed by 35 cycles of 95°C 30s, 56°C 30s, 72°C 1 min). Screening was also conducted for additional symbiotic bacteria. The list of these primers, protocols and results can be found in the supporting information (Supplementary Tables S2, S3). PCR’s were conducted using 2 μL of the extracted genomic DNA, Illustra PuReTaq Ready-To-Go PCR beads (GE Healthcare), 1 μL each of 10 μM primers listed above and 21 μL of water. PCR products were run on agarose gels and visualized using GelGreen (Biotium) nucleic acid stain to verify whether amplification of the correct gene fragment had taken place. PCR products destined for sequencing were cleaned using the QIAquick PCR purification Kit (Qiagen), and concentration of DNA was measured using a Nanodrop ND-1000 spectrophotometer (Thermo Scientific). PCR products were sequenced using 20 μL reactions containing 3 μL of Big Dye v3.1 (Applied Biosystems), 1.6 μL of 2 μM primer and variable amounts of DNA and water depending on the PCR product concentration. Amount of DNA to be used in a sequencing reaction was calculated based on 5 ng of DNA per 100 bp of PCR product to be sequenced. Sequencing reactions were cleaned using PERFORMA® Ultra 96-Well Plate (Edge Bio, Gaithersburg, Maryland) and run on the Applied Biosystem 3730xl DNA Analyzer (Life Technologies) at the University of Illinois Keck Center. Sequences were analyzed using the Sequencher® v5.0 (Gene Codes Inc., Ann Arbor, Michigan) and manually aligned using PAUP version 4.0.

### Individual based population model

We developed a simple population model in SAS IML (Supplementary material 1a, b) which modeled the greenhouse and growth chamber whole plant experiments using the individual leaf experiment data. As with the whole plant experiment, each model run began with 20 individuals and ran for 14 days. Data for each individual in the model were randomly selected from the individual fecundity curves of the specific aphid biotype grown on the respective soybean variety used in the individual leaf experiment. Each day of the model, the number of individuals produced (sum of all individuals produced for the day) was determined and that number of individuals randomly selected and added as new individuals in the model starting at that day of the model run. Missing values in the matrix represent points when the aphid is no longer alive or was not yet added to the model. Zero values were used to indicate that an individual is present but not producing offspring. The number of individuals was calculated as the number of non-missing values for a given day. The model was run 3,000 times with all five aphid biotypes and four soybean varieties.

This simple model incorporates the cumulative impact of all aspects of the fecundity curves to produce an idealized fecundity rate based upon optimal conditions and does not include intraspecific competition. The model therefore isolates the impact of the different soybean varieties on aphid population growth.

## Analysis methods

### Whole plant—growth chamber and greenhouse experiments

Prior to analysis, aphid population counts were transformed by adding one and taking the log base 10 to correct for non-constant variance among the treatments. Variance homogeneity tests indicated that the variance between the two trials were not significant, therefore data from both trials were combined in the final analysis.

In the greenhouse experiment, cages were set up in pairs as part of a randomized complete block design. Cage pair was therefore used as a random factor in the greenhouse model. Tukey–Kramer adjustments were used for post-hoc analyses.

Several methods were tried to transform the count data from the fecundity experiment conducted in the growth chamber and greenhouse. The data were overdispersed when analyzed using a Poisson model, therefore, we used a generalized linear model (Proc Genmod, SAS ver. 9.4) with a negative binomial distribution and a log link function as suggested by Agresti (2002).

### Detached leaves experiment

We characterized overall fecundity (the number of aphid nymphs deposited by individual aphids over the duration of the experiment), as well as the pattern of fecundity (day first nymph deposited, maximum number of nymphs deposited in a day, and day maximum number of nymphs were deposited), and survival rate. We also tested whether there was a pattern of variability in fecundity among the different aphid strains on the different soybean genotypes.

For the four measures that were analyzed, (1) total number of nymphs, (2) maximum number of nymphs, (3) day of first nymph, and (4) the day of maximum nymphs deposited, transformations were unsuccessful at normalizing the data or reducing hetero-skedasticity. For the same measures listed above, if the interaction term of soybean variety by aphid strain was not found to be significant, the analysis was rerun without the interaction term.

### Detached leaves: aphid fecundity—total nymphs deposited per aphid

A generalized linear model (Proc Genmod, SAS ver. 9.4) with a negative binomial distribution and a log link function as suggested by Agresti (2002) was used. Tukey–Kramer post-hoc analyses were used to compare least square means.

### Detached leaves: characterization of fecundity—maximum nymphs deposited

A weighted least squares approach, modeling the mean response, was used for the analysis (Proc Catmod, SAS ver. 9.4). Contrasts compare the maximum number of aphids deposited on the susceptible *W82* soybean genotype to each of the three other genotypes. Contrasts also compared lab aphid strains Bt1 (*A*+*W*−) to Bt3 (*A*+*W*+), as well as each of these strains to their derived antibiotic treated strains: Bt1 (*A*+*W*−) to and Bt1 (*A*−*W*−) and Bt3 (*A*+*W*+) to Bt3 (*A*−*W*−) and Bt3 (*A*−*W*+), and the two latter antibiotic treated strains to each other. A Bonferroni adjustment was used to determine alpha for multiple comparisons.

### Detached leaves: characterization of fecundity—day first nymph deposited

A weighted least squares approach was not an acceptable analysis for comparing the first day that aphids were deposited due to a problem with linear dependence. Cochran–Mantel–Haenszel row mean score (Proc Freq, SAS ver. 9.4) was therefore used to assess the effect of soybean genotype and aphid strain on first day of nymph deposition. If an aphid did not deposit nymphs, it was removed from the analysis.

### Detached leaves: characterization of fecundity—day maximum number of nymphs deposited

We analyzed the day that the maximum number of nymphs were deposited using a generalized linear model (Proc Genmod, SAS ver. 9.4) with a negative binomial distribution with an identify link function. The Poisson distribution resulted in an overdispersed model and a log link resulted in failure of the relative Hession convergence criteria. Tukey–Kramer post-hoc analyses were used to compare least square means.

### Detached leaves: survival analysis

Two models were used to assess the effect of soybean genotype on the survival of different strains of aphids. A logistic regression event/trials model via Proc Logistic (SAS ver. 9.4) was used to model the effect of survival to day 14. In addition, Proc Phreg (SAS ver 9.4) was used to model survival functions over the duration of the study. In both cases non-significant interactions and variables were removed until only significant factors were left in the model.

### Detached leaves: variability of fecundity

We used a model II ANOVA to determine whether soybean genotype had an impact on the variability of the total number of nymphs produced (Proc Nested, SAS ver. 9.4). Only aphids that survived to the end of the experiment were included in the analysis. The analysis does not allow for unequal sample size, thus a random sample of seven samples from each aphid strain/genotype combination was used in the analysis. The analysis was repeated 10 times, with a different selection of samples, to ensure that results were consistent. The *p*-values presented are averages of the *p*-values from the 10 runs. Because post-hoc tests are limited in model II ANOVAs, a set of tests were run comparing Bt1 (*A*+*W*−) to Bt3 (*A*+*W*+), Bt1 (*A*+*W*−) to Bt1 (*A*−*W*−), and Bt3 (*A*+*W*+) to each of its antibiotic-treated sub-strains Bt3 (*A*−*W*+) and Bt3 (*A*−*W*−). Comparisons were also made between the susceptible *W82* genotype and each of the resistant genotypes (*Rag1*, *Rag2*, *Rag1 + 2*) using only data from Bt1 (*A*+*W*−) and Bt3 (*A*+*W*+). As with the overall analyses, 10 runs each, using seven random samples were used for the post-hoc tests and the *p*-value was averaged over all runs. The alpha used in these analyses was Bonferroni adjusted to account for multiple tests.

### Individual based population model

We ran the same ANOVA model used for the whole plant experiments for successive sets of three models representing the three replicates used in the greenhouse and growth chamber experiments (each run was randomly selected so successive model selection is still random). We calculated the average *p* value for each main effect and interaction for the 1,000 model experiments.

## Results

### Reproduction on whole plants

**Figure 1:**
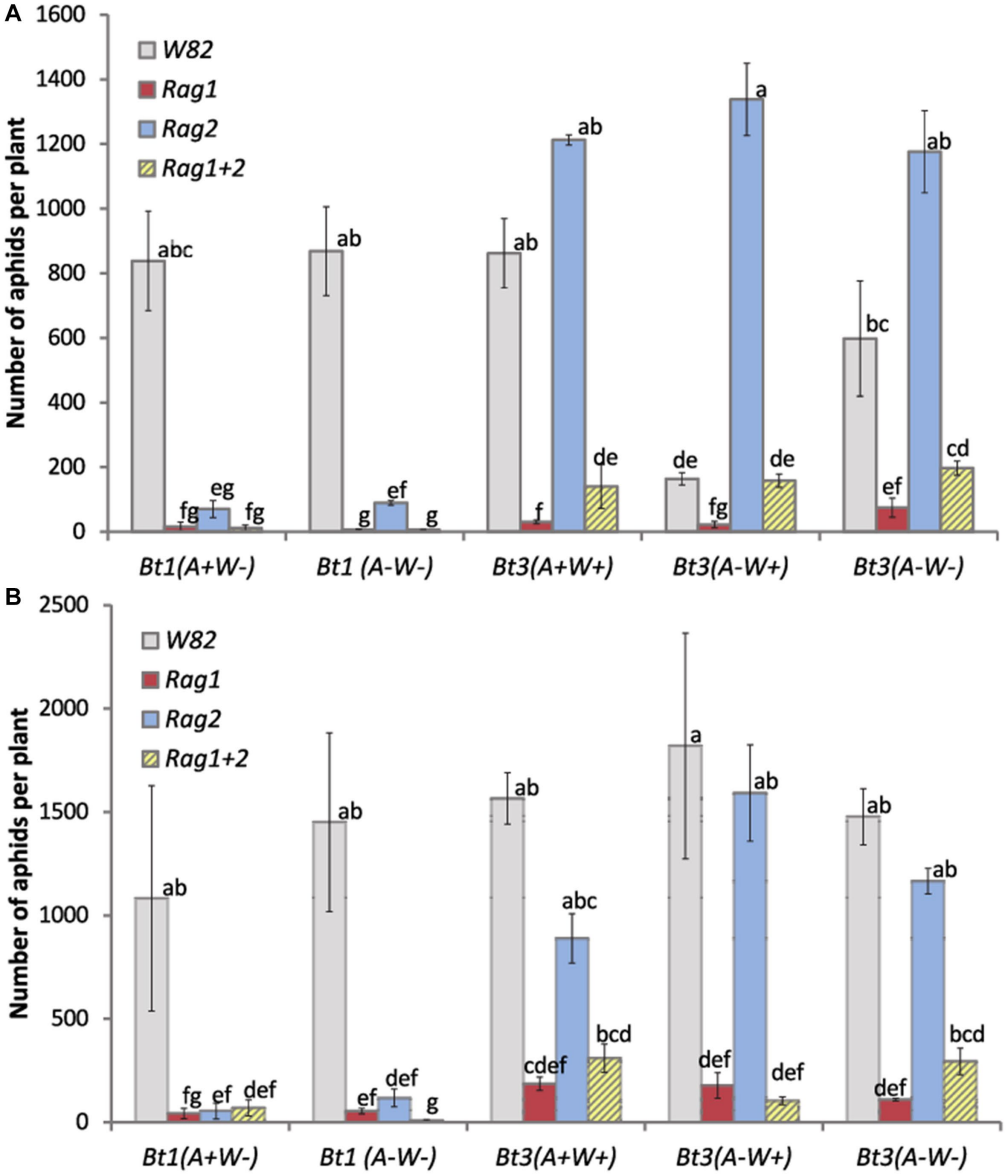
Both aphid strain and soybean genotype affected aphid population size in whole plant experiments. Average number of aphids per plant for 5 clonal strains of aphids [Bt1(*A*+*W*−), Bt1(*AW*−), Bt3(*A*+*W*+), Bt3(*A*−*W*+), Bt3(*A*−*W*−)], grown on caged whole plants in a greenhouse **(A)** and an environmental chamber **(B)** on 4 genotypes of soybean (*W82*, *Rag1*, *Rag2*, *Rag1 + 2*). Counts were made at the end of the experiment. Data were analyzed with a general model using a negative binomial distribution and a log link function. There was a significant interaction between aphid strain and soybean genotype for the greenhouse (df = 12, *X^2^* = 22.53, *p* = 0.0320) and environmental chamber experiments (df = 12, *X^2^* = 39.49, *p* < 0.0001). Means with the same letter are not significantly different using Tukey–Kramer *post hoc* comparisons (*p* < 0.05). The study included three replicates of each aphid strain/soybean variety combination for both experiments.

There was a significant interaction between aphid strain and soybean genotype for the greenhouse (df = 12, *X^2^* = 22.53, *p* = 0.0320) and environmental chamber experiments (df = 12, *X^2^* = 39.49, *p* < 0.0001), demonstrating aphid biotype specificity toward plant host genotypes. In both experiments, all strains of Bt3 had significantly higher aphid counts than the two strains of Bt1 (Figure 1) when grown on *Rag2*.

The two Bt1 strains, (*A*+*W*−) and (*A*−*W*−), behaved similarly, with reduced fecundity on all resistant genotypes, the three Bt3 strains, (*A*+*W*+), (*A*−*W*−), (*A*−*W*+), also behaved similarly to each other with higher fecundity on *W82* and *Rag2* and lower fecundity on *Rag1* and *Rag1 + 2* (Figure 1). There was no significant difference in the performance of all three Bt3 strains on *Rag1* (letter f), *Rag 2* (letter a), or *Rag 1 + 2* (letter d); likewise, there was no significant difference for Bt1 strains on *Rag1*, *Rag1 + 2* (letter g), or *Rag 2* (letters e, f) (Figure 1). In the greenhouse experiment, Bt3 (*A*−*W*+) had significantly lower aphid counts on *W82* plants (Figure 1A), and all three plants were severely damaged by aphid feeding as compared to the other plants at the end of the experiment. The three plants in this latter treatment were, respectively, classified as very poor, poor, and fair (using the non-parametric plant health scale referred to in the methods) at the end of the experiment. No other treatment in the greenhouse had all three plants classified as poor or fair. The susceptible *W82* genotype in the environmental chamber did not have such decrease in aphid numbers (Figure 1B). **T**here was not a significant difference attributable to the presence or absence of *Wolbachia* or *Arsenophonus* in the aphids.

### Detached leaves: fecundity of individual aphids

**Figure 2:**
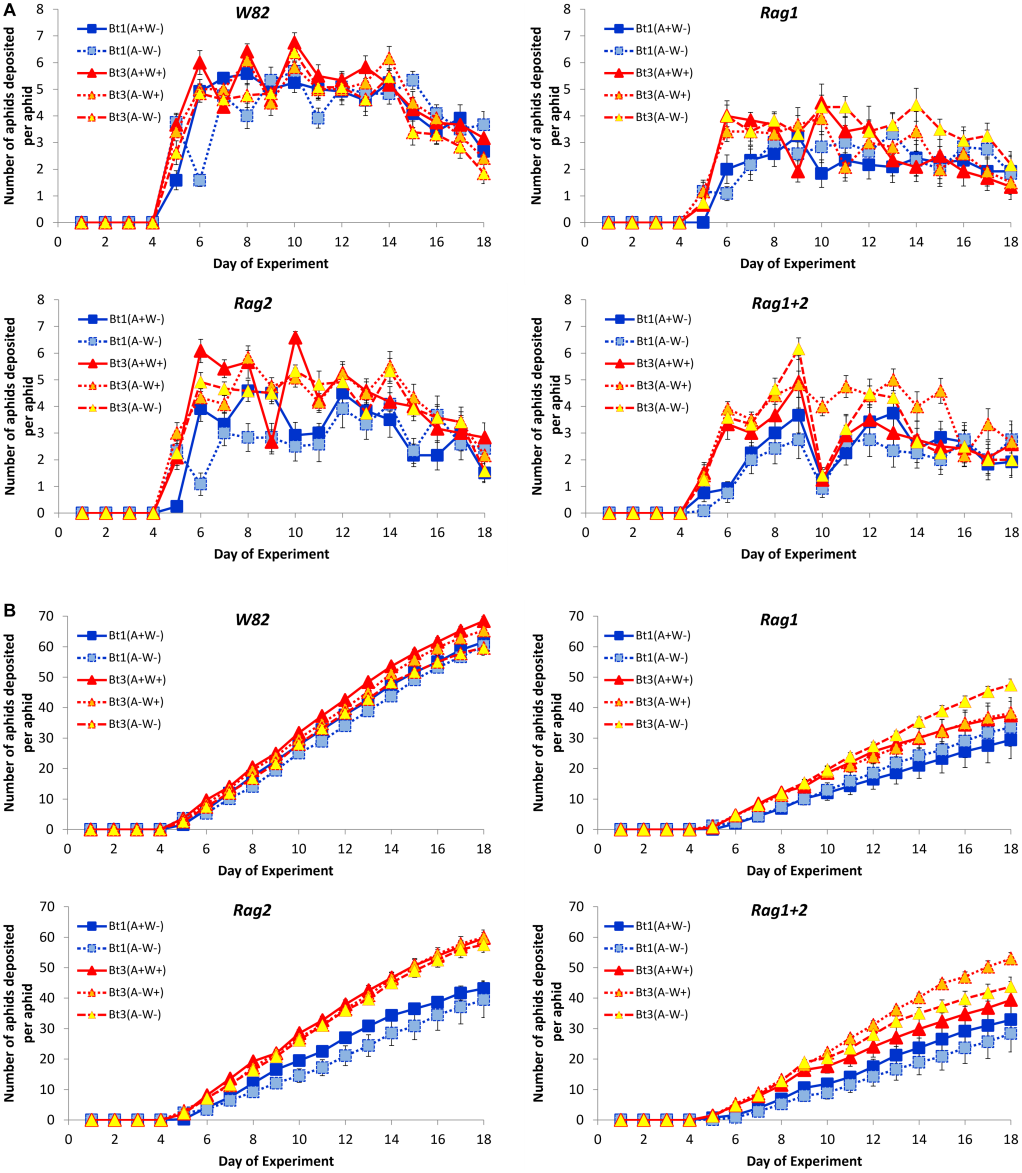
Pattern of cumulative and daily aphid nymph deposition by individual aphids on detached leaves. Average number **(A)** and cumulative number **(B)** of aphids deposited per day by strain for individual aphids grown on detached leaves of four soybean genotypes (*W82*, *Rag1*, *Rag2*, and *Rag1 + 2*). Error bars are standard errors of the average number of aphid nymph deposited. Characterizations of the fecundity curves are described in the text.

Average number and cumulative number of nymphs deposited per day per genotype leaf over the duration of the experiment (Figure 2) were characterized by the overall fecundity (the number of nymphs deposited by individual aphids for the duration of the experiment); pattern of fecundity (maximum number of nymphs deposited in a day, day first nymph deposited, and day maximum number of nymphs were deposited); and survival rate.

### Detached leaves: overall fecundity

**Figure 3:**
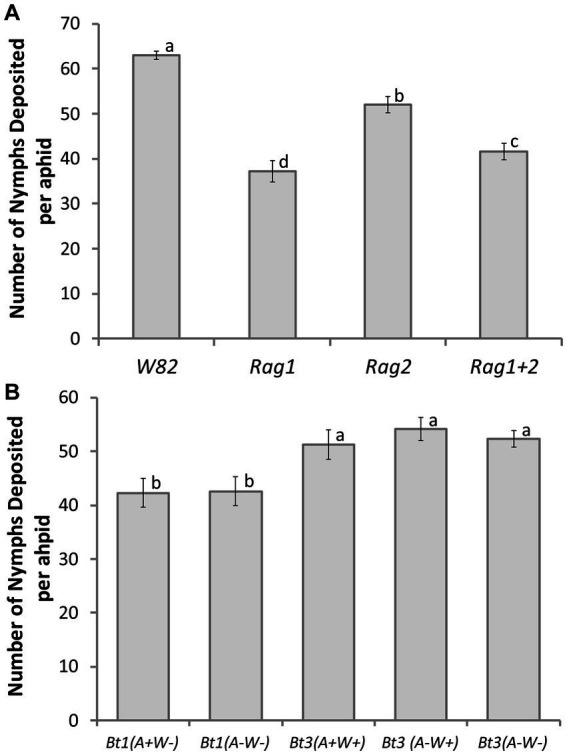
Aphid strain and soybean genotype affected number of aphid nymphs deposited on detached leaves. Average number of nymphs deposited per aphid over a 14-day period for 5 clonal strains of aphids [Bt1(*A* + *W*−), Bt1(*A*−*W*−), Bt3(*A* + *W*+), Bt3(*A*−*W*−), Bt3(*A*−*W*−)] cultured on detached leaves of 4 soybean genotypes (*W82*, *Rag1*, *Rag2*, *Rag1 + 2*). Data presented by soybean genotype **(A)** and aphid strain **(B)**. Data were analyzed with a general model using a negative binomial distribution and a log link function. Soybean variety was a significant factor in the number of nymphs deposited per aphid (*X^2^* = 48.33, df = 3, *p* < 0.0001) as was aphid strain (*X^2^* = 15.21, df = 4, *p* = 0.0043). Means with the same letter are not significantly different using Tukey–Kramer *post hoc* comparisons (*p* < 0.05). The study included 12 replicates of each aphid strain/soybean genotype combination except for Bt3 (*A*−*W*−) on *W82* soybean which had 13 replicates.

The interaction between aphid clonal strain and soybean genotype was not significant (df = 12, *X^2^* = 10.32, *p* = 0.5883); however, both main effects were significant (soybean genotype *X^2^* = 48.33, df = 3, *p* < 0.0001; aphid strain *X^2^* = 15.21, df = 4, *p* = 0.0043). In Tukey–Kramer *post hoc* tests fecundity on all four soybean genotypes were significantly different from each other (Figure 3A). The susceptible *W82* plants had the highest number of aphid nymphs deposited (63 ± 0.94 nymphs deposited), while the *Rag1* plants had the lowest number of nymphs deposited over the same time-period (37 ± 2.32 nymphs deposited). The two Bt1 strains had significantly lower fecundity than the three Bt3 strains. The pattern of overall fecundity, with the susceptible genotype (*W82*) having the highest and *Rag1* the lowest number, was consistent across all aphid strains (Figure 3A); however, Bt1 (*A*+*W*−) and Bt1 (*A*−*W*−) deposited significantly fewer nymphs on all genotypes compared to the three Bt3 strains on the same genotypes (Figure 3B). The cumulative number of nymphs deposited between Bt1 and Bt3 strains on *Rag2* can be observed in Figure 2B. The presence or absence of *Wolbachia* and *Arsenophonus* did not significantly affect fecundity within the two Bt1 and three Bt3 aphid strains (Figure 3B).

### Detached leaves: pattern of fecundity—maximum aphids deposited

**Figure 4:**
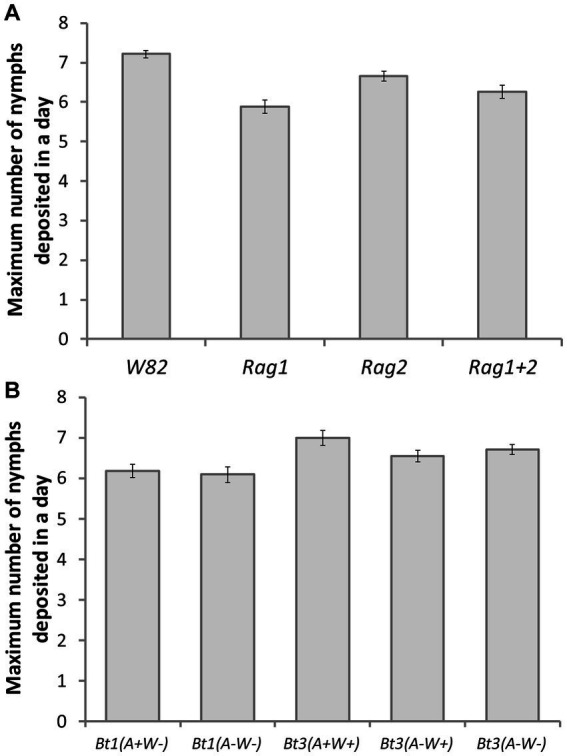
Aphid strain and soybean genotype affected maximum number of aphid nymphs deposited on detached leaves. Average number of the maximum number of nymphs deposited by an individual aphid in a single day: by genotype **(A)** and by aphid strain **(B)**. There were significant differences among genotypes (df = 3, *X^2^* = 92.83, *p* < 0.001) and aphid strains (df = 4, *X^2^* = 46.79, *p* < 0.0001). Using Bonferroni adjusted tests of specified contrasts of genotypes and strains (α = 0.0063 see methods for more information), aphids had a higher maximum number of nymphs deposited in a day on the susceptible genotype (*W82*) than the other genotypes. Bt1(*A*+*W*−) was significantly lower than Bt3(*A*+*W*+) but not from Bt1(*A*−*W*−). Bt3(*A*+*W*+) was significantly higher than Bt3(*A*−*W*+) and Bt3(*A*−*W*−).

For the maximum number of nymphs deposited, the interaction term of soybean genotype by aphid strain was not significant (df = 12, *X^2^* = 19.26, *p* = 0.08214) and was therefore removed, prior to running the main effects model. Both soybean genotype (df = 3, *X^2^* = 92.83, *p* < 0.001) and aphid strain (df = 4, *X ^2^* = 46.79, *p* < 0.0001) were significant. A total of eight contrasts were tested in this experiment resulting in a Bonferroni adjusted alpha level of 0.0063. Aphids grown on the susceptible genotype (*W82*) had a higher maximum number of nymphs deposited than those cultured on any resistant genotype (*Rag1*, *Rag2*, *Rag1 + 2*) (Figure 4A). There was not a significant difference between Bt1 (*A*+*W*−) and Bt1 (*A*−*W*−), but Bt1 (*A*+*W*−) had significantly lower maximum number of nymphs deposited than Bt3 (*A*+*W*+) (Figure 4B). Bt3 (*A*+*W*+) had significantly higher maximum number of nymphs deposited than either Bt3 (*A*−*W*+) or Bt3 (*A*−*W*−), but Bt3 (*A*−*W*+) and Bt3 (*A*−*W*−) were not significantly different from each other. *Arsenophonus* significantly impacted Bt3 but not Bt1.

### Detached leaves: characterization of fecundity—day first nymph deposited

There was a significant difference in the day that the first nymph was deposited (Cochran–Mantel–Haenszel row mean scores differ df = 4, *X^2^* = 25.23, *p* < 0.0001). When on the *W82* plant genotype all aphid strains deposited nymphs earlier than on resistant genotypes (Table 1). The Bt1(*A*+*W*−) strain deposited nymphs later than the other strains on all plant genotypes except for *Rag1 + 2* genotype where Bt1(*A*−*W*−) was the most delayed. *Arsenophonus* impacted the day of first nymph deposition in Bt1 but not Bt3.

**Table 1:** Day nymphs were first deposited by aphid strain on soybean genotypes.

		Day first nymph deposited^a^			
Soybean genotype	Strain	Day 5	Day 6	Day 7	Average
*W82*	Bt1(*A*+*W*−)	8	4	0	5.3(±0.1)
	Bt1(*A*−*W*−)	12	0	0	5(±0)
	Bt3(*A*+*W*+)	11	1	0	5.1(±0.1)
	Bt3(*A*−*W*+)	12	0	0	5.2(±0.1)
	Bt3(*A*−*W*−)	10	3	0	5(±0)
*Rag1*	Bt1(*A*+*W*−)	0	8	1	6.1(±0.1)
	Bt1(*A*−*W*−)	6	4	0	5.4(±0.2)
	Bt3(*A*+*W*+)	6	5	1	5.6(±0.2)
	Bt3(*A*−*W*+)	7	5	0	5.6(±0.1)
	Bt3(*A*−*W*−)	5	7	0	5.4(±0.1)
*Rag2*	Bt1(*A*+*W*−)	2	10	0	5.8(±0.1)
	Bt1(*A*−*W*−)	9	1	0	5.1(±0.1)
	Bt3(*A*+*W*+)	9	3	0	5.3(±0.1)
	Bt3(*A*−*W*+)	11	1	0	5.3(±0.1)
	Bt3(*A*−*W*−)	9	3	0	5.1(±0.1)
*Rag1 + 2*	Bt1(*A*+*W*−)	4	7	0	5.6(±0.2)
	Bt1(*A*−*W*−)	0	8	4	6.3(±0.1)
	Bt3(*A*+*W*+)	7	5	0	5.4(±0.1)
	Bt3(*A*−*W*+)	7	5	0	5.5(±0.2)
	Bt3(*A*−*W*−)	6	6	0	5.4(±0.1)

Numbers in table represent the count of aphids that began depositing on a particular day of the experiment as well as the average.

a There was a significant difference in the day the first nymph was deposited, (Cochran–Mantel–Haenszel row mean scores differ df = 4, χ^2^ = 25.23, *p* < 0.0001).

### Detached leaves: characterization of fecundity—day maximum number of nymphs were deposited

**Figure 5:**
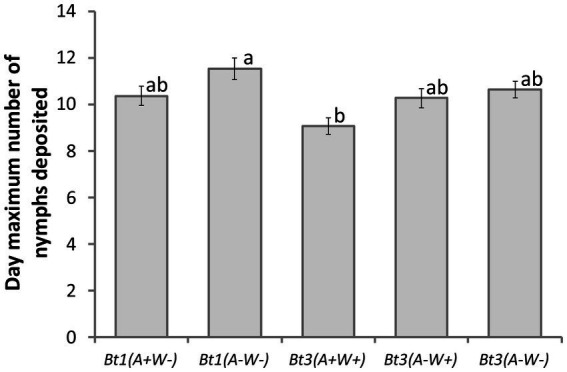
Aphid strain affected the day maximum number of aphid nymphs were deposited on detached leaves. Average day that the maximum number of nymphs were deposited by five aphid strains on four soybean genotypes (df = 4, *X^2^* = 14.13, *p* = 0.0069). Error bars are standard errors of the means. Means with the same letter are not significantly different using Tukey–Kramer *post hoc* comparisons (*p* < 0.05). Aphids that died before depositing nymphs were not included in the analysis. See Table 1 for sample size.

Bt3(*A*+*W*+) was the first strain to deposit the maximum number of nymphs in a day, while Bt1(*A*−*W*−) was the last, but there was not a significant difference within the Bt1 or Bt3 strains (Figure 5) (Aphid strain effect df = 4, *X^2^* = 14.13, *p* = 0.0069). Neither soybean genotype nor the interaction was significant (strain x genotype interaction df = 12, *X^2^* = 17.93, *p* = 0.1179; genotype df = 3, *X ^2^* = 1.53, *p* = 0.6752). *Arsenophonus* and *Wolbachia* did not impact the day maximum number of nymphs were deposited.

### Detached leaves: survival analysis

**Figure 6:**
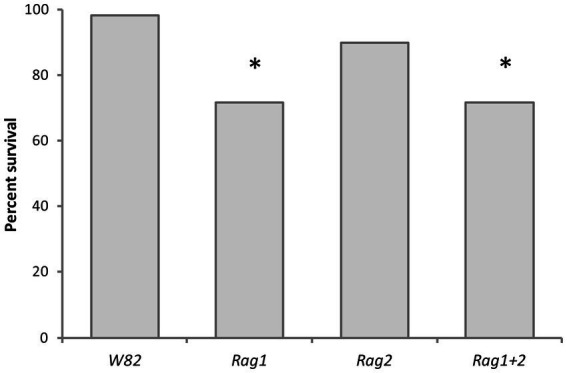
Resistant soybean genotypes decreased aphid survival on detached leaves. Percent aphid survival at day 14, cultured on leaves of four varieties of soybean (*W82*, *Rag1*, *Rag2*, *Rag1 + 2*). The percentages shown were calculated by combining all five strains of aphid used in the study [Bt1(*A*+*W*−), Bt1(*A*−*W*−), Bt3(*A*+*W*+), Bt3(*A*−*W*+), Bt3(*A*−*W*−)]. Asterisks indicate a significant difference (*p* < 0.05) from the susceptible *W82* soybean genotype. Aphids survived significantly better (*p* < 0.05) on the *W82* variety of soybean than on genotypes with *Rag1* or *Rag1 + 2* (*X^2^* = 15.3293, df = 3, *p* = 0.0016). There was not a significant difference between *W82* and *Rag2*.

Aphid strain had no effect on survival, but soybean genotype was a significant factor (proc logistic *X*^2^ = 15.3293, *p* = 0.0016; Proc Phreg Wald *X*^2^ = 14.70, df = 3, *p* = 0.0021). Aphid strains had significantly lower survival rates on *Rag1* and *Rag1 + 2* compared to the susceptible genotype (*W82*). Survival rates and functions of aphids for all strains on *Rag2* were not significantly different from aphids on the *W82* genotype (Figure 6). *Arsenophonus* and *Wolbachia* did not impact survival of aphids.

### Detached leaves: variability of fecundity

**Figure 7:**
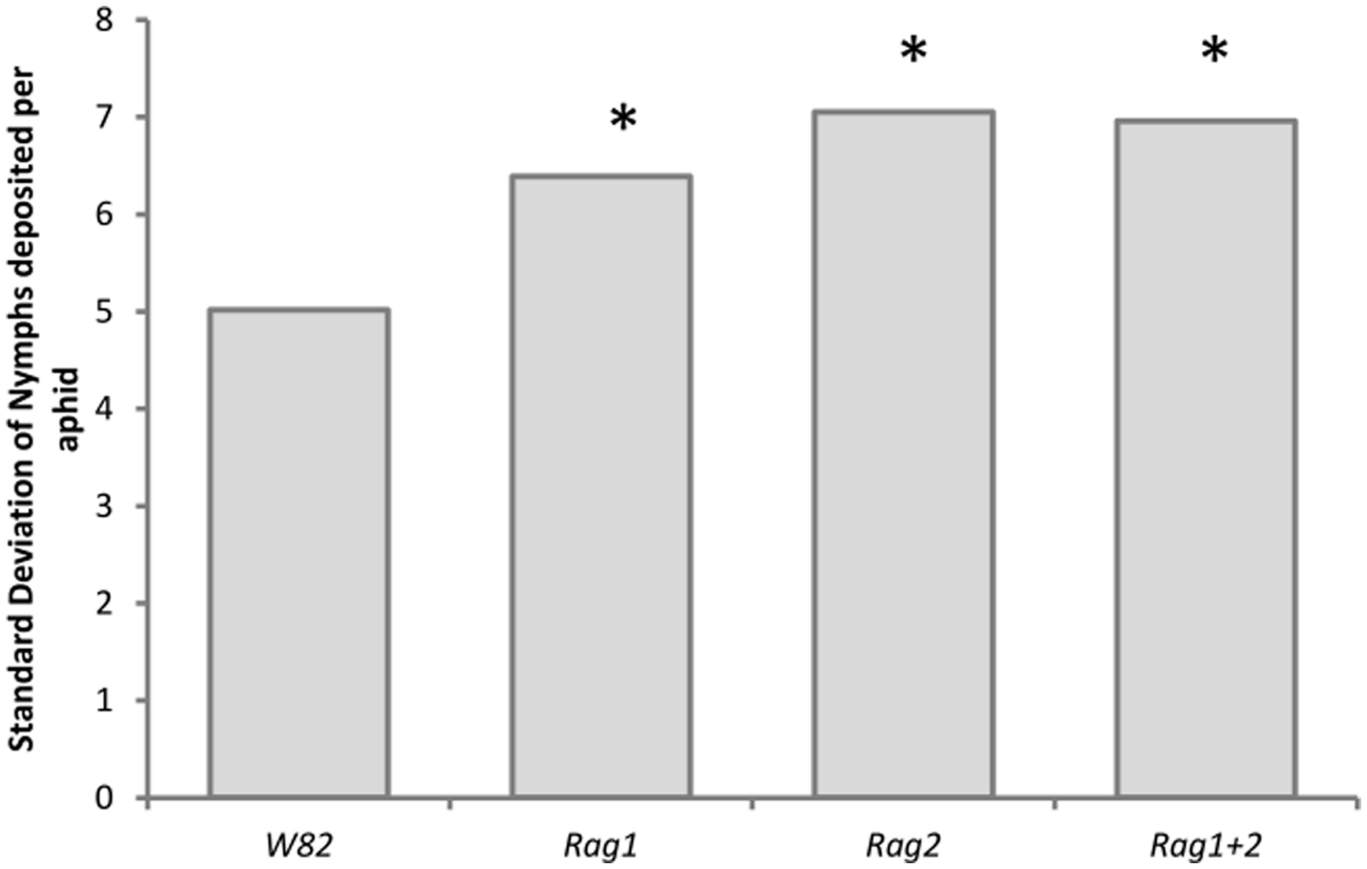
Resistant soybean genotypes increased variability of total fecundity per aphid on detached leaves. Standard deviation of the total number of nymphs deposited by aphids that survived to the end of the experiment (18 days). The standard deviation was calculated across all five strains for each plant genotype. There is a significant difference in the variation of nymphs deposited using model II ANOVA testing for significant difference of variation (df = 3, *p* < 0.0001). Asterisks indicate a significant difference (*p* < 0.05) from the *W82* soybean genotype. *W82* genotype was significantly less variable that *Rag1, Rag2* or *Rag1 + 2*.

There was a significant difference in the variability of total fecundity per aphid on the different genotypes (df = 3, *p* < 0.0001) but not among aphid strains (df = 4, *p* < 0.05). The analysis only included aphids that survived the entire experiment (18 days, 7 aphids per aphid strain, soybean genotype combination, see methods for more information on sample size), providing a conservative measure of variability. Aphids cultured on the susceptible genotype (*W82*) had less variability in the total number of nymphs deposited when compared to the resistant *Rag* soybean genotypes (Figure 7). No comparisons were made among the resistant genotypes. *Arsenophonus* and *Wolbachia* did not impact variability of fecundity.

### Individual based population model

**Figure 8:**
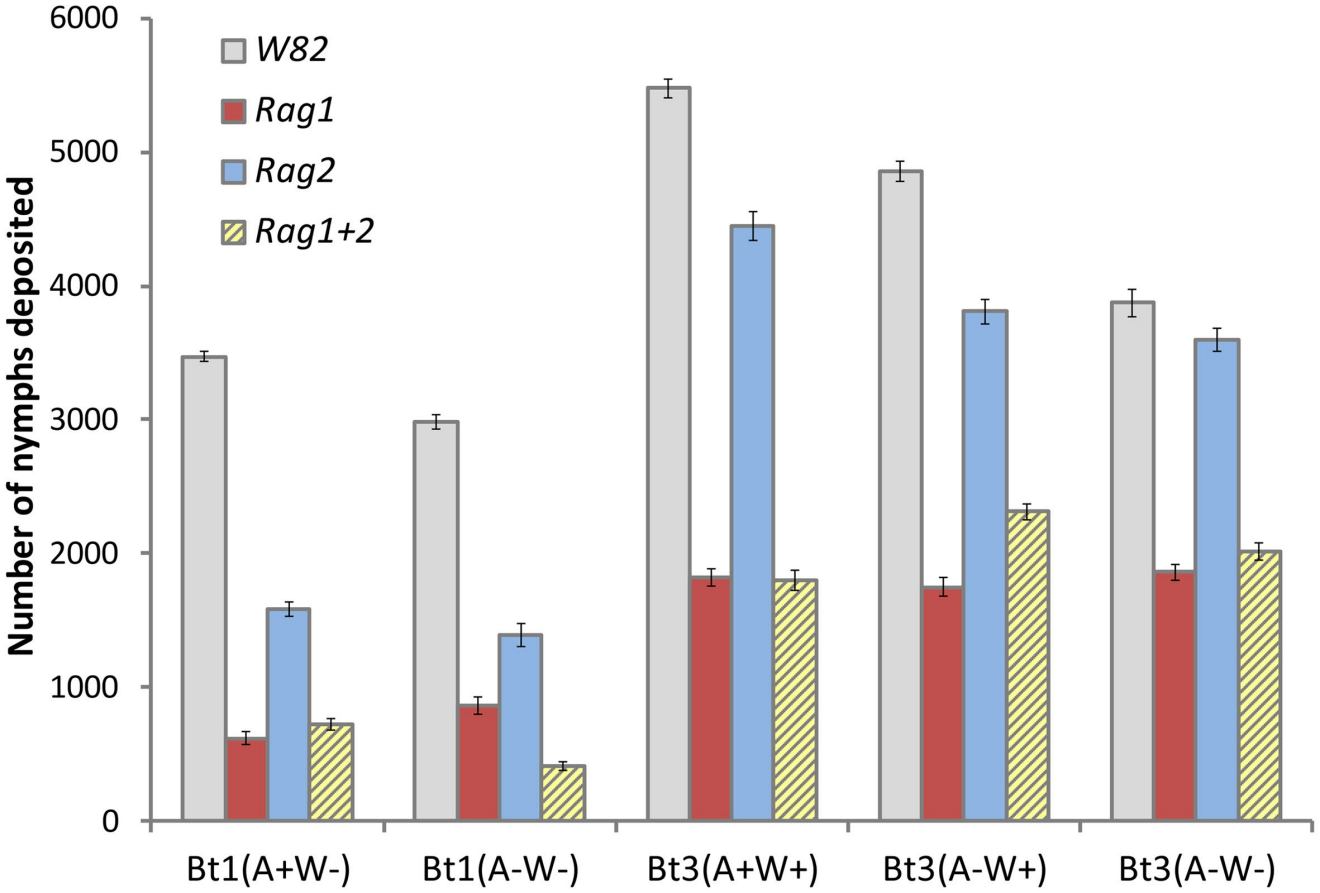
Simulations using individual leaf data found significant interactions similar to those in whole plant experiments. Average number of aphids per plant from simulations of whole plant experiment using individual leaf data. The study simulated five clonal strains of aphids [Bt1(*A*+*W*−), Bt1(*A*−*W*−), Bt3(*A*+*W*+), Bt3(*A*−*W*+), Bt3(*A*−*W*−)], grown on four genotypes of soybean (*W82*, *Rag1*, *Rag2*, *Rag1 + 2*) in each of 1,000 simulated experiments. The average represents the average of the average number of aphids per plant and error bars represent the average standard error. Using the same statistical method as used in the whole plant experiment, there was a significant interaction effect in more than 99% of the model runs.

**Figure 9:**
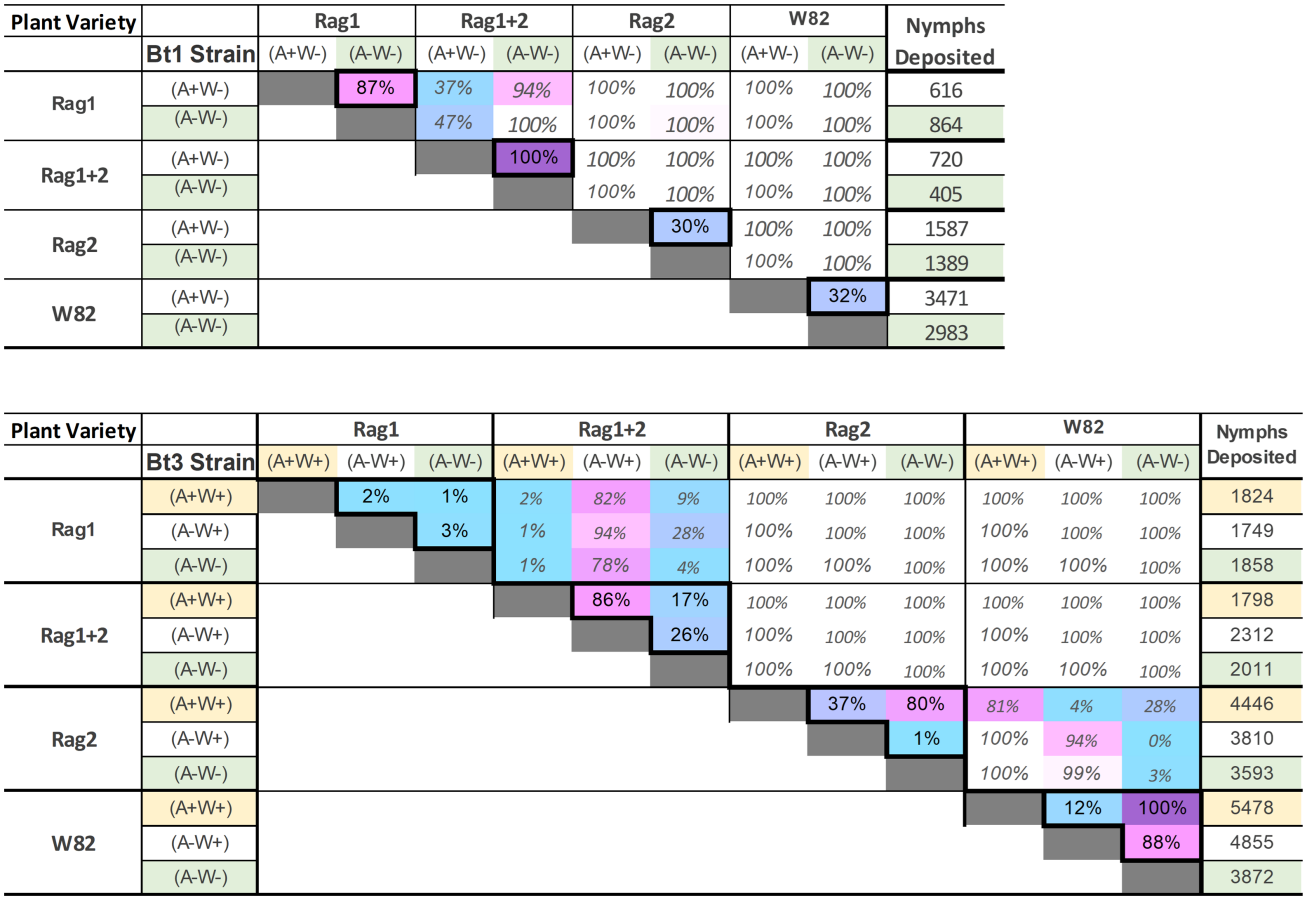
Proportion of model runs with significant differences (*p* < 0.05) among Bt1 (top) and Bt3 (bottom) biotype strains for all soybean varieties. Differences outside the diagonal, comparing results on the same soybean variety, are grayed out. Comparisons with a larger proportion of significant differences are purple/pink, those with a lower proportion are pale blue. Differences of 100% only occurred outside the diagonal and were left white.

The overall pattern of the model runs (Figure 8) illustrates a pattern similar to that observed in the greenhouse and growth chamber experiments. In contrast to the analyses of the individual aphid data outside the model, there was a significant interaction effect between soybean variety and aphid strain (*p*-value was <0.001 for both the main effects and interaction in more than 99% of model experiments). The *Rag1* and *Rag 1 + 2* genotypes had a stronger impact than *Rag2*, and Bt1 strains are more strongly impacted by *Rag1*, *Rag1 + 2* and *Rag2* than are Bt3 strains. For the Bt1 strain, which does not have *Wolbachia*, *Arsenophonus* decreased fecundity on *Rag1* (100% of models significant at *p* < 0.05, diff = 720–402 = 315) and increased total fecundity on *Rag1 + 2* (87% of models significant at *p* < 0.05, diff = 864–616 = 248) (Figure 9A). *Arsenophonus* had no significant effect when Bt1 strain aphids are exposed to *Rag2* and *W82*.

For the Bt3 strain, neither *Wolbachia* nor *Arsenophonus* impacted fecundity on *Rag1*, however, *Arsenophonus* decreased fecundity on *Rag1 + 2* (87% of models significant at *p* < 0.05 (*A*−*W*+) vs. (*A*+*W*+)*,* diff = 2,312–1,798 = 514) (Figure 9B). Fecundity of the Bt3 strain increased on Rag2 if both *Wolbachia* and *Arsenophonus* were present [80% of models were significant at *p* < 0.05 (*A*+*W*+) vs. (*A*−*W*−), diff = 4,449–3,593 = 853]. For the Bt3 strain when on W82, the presence of *Wolbachia* (whether alone or with *Arsenophonus*) resulted in higher fecundity than when *Wolbachia* was absent [100% (*A*+*W*+) vs. (*A*−*W*−) and 88% (*A*−*W*+) vs. (*A*−*W*−) models significant at *p* < 0.05, diff (*A*+*W*+) = 5,478–3,872 = 1,606, diff (*A*−*W*+) = 4,855–3,872 = 983].

## Discussion

We did not detect differences in overall fecundity in *A. glycines* with or without *Arsenophonus* or *Wolbachia*, in experiments using whole plants or individual aphids, but did observe differences in patterns of fecundity of individual aphids on detached leaves. We also observed differences in overall aphid fecundity when individual aphid data was used in simulations of whole plant experiments (Table 2, Figure 9). We were able to detect these differences in the simulations because measurements of individual aphids on detached leaves removed the impact of intraspecific competition and plant health which confounded the whole plant experiments.

**Table 2:** Significant measures of *Arsenophonus* and *Wolbachia* fecundity on soybean aphid reproduction by experiment.

Experiment	Measure	*Arsenophonus*	*Wolbachia*	*Arsenophonus* + *Wolbachia*
Detached/individual leaf				
	Maximum number of aphids deposited	↑ Bt3 increased (on all plant varieties)		
	Day first aphid deposited	↓Bt1 deposit later (on Rag1, Rag2, W82)		
individual based model				
	Bt1 effects	↓ decreased on Rag1, ↑ increased on Rag1 + 2		
	Bt3 effects	↓ decreased on Rag1 + 2	↑ increased on W82	↑ increased on Rag2

Only significant results are included in the table. Results from the whole plant study and some measures from the detached leaf/individual leaf study did not have significant effects and thus were not included in the table.

On whole plants (Figure 1), Bt1 and Bt3 aphid strains produced levels of offspring matching those expected from previous published work; *Rag1* and *Rag1 + 2* plants reduced total aphid counts for all Bt1 and Bt3 strains, but *Rag2* plants only reduced counts of Bt1 strains, due to Bt3 being virulent on *Rag2* (Hill et al., 2010). As indicated earlier, there were no detectible differences due to the presence of bacterial symbionts when aphids were grown on whole plants.

The average and cumulative number of nymphs deposited for all Bt1 and Bt3 strains decreased when aphids were placed on resistant genotypes but were most pronounced for both strains when placed on *Rag1* and *Rag1 + 2* detached leaves. These results indicate that detached leaves of these resistant genotypes retained their resistance. As in the whole plant experiments, we were not able to determine differences in overall fecundity due to presence or absence of bacterial symbionts in overall fecundity of individual aphids on detached leaves, however, we were able to detect differences due to bacterial symbionts in simulations based on the individual plant study. Aphid counts in whole plant studies combine the impact of survival and fecundity of individual aphids, intraspecific competition, and the impact of aphid population size on plant health. In contrast, our observations of individual aphids on detached leaves permitted the separation of fecundity and survival patterns from the influence of competition or plant health. When the differences observed in individual aphids are combined through simulations, the individual differences were magnified, and this allowed the detection of differences among aphid populations and observed differences that exceeded the whole plant studies.

On detached leaves, secondary symbionts impacted the maximum number of nymphs deposited (Figure 4), the day the first nymph was deposited (Table 1) and may have delayed the peak deposition of aphids (Figure 5). However, the bacteria impacted Bt1 and Bt3 aphid strains differently (Table 2, Figure 9). *Arsenophonus* when together with *Wolbachia*, increased the maximum number of nymphs deposited by Bt3 strains. In Bt1, which does not have *Wolbachia*, there was not an increase. This increase with both *Arsenophonus* and *Wolbachia* present in max aphids deposited in a day may have resulted in the increased fecundity observed on *Rag2* for Bt3(*A*+*W*+) in the individual based model results. In addition, *Wolbachia* increased overall fecundity in the individual based models when Bt3 was reared on *W82*. In the Bt1 strains, the presence of *Arsenophonus* only impacted the first day of deposition, which was delayed when aphids were placed on *Rag1*, *Rag2,* and *W82.* When Bt1 aphids without *Arsenophonus* were reared on *Rag1 + 2,* first day of deposition was slightly delayed compared to the Bt1 strain with *Arsenophonus* (Table 1). In the individual based model, day of deposition was a probable cause for the decreased fecundity on *Rag1* and increased fecundity on *Rag1 + 2* for Bt1 aphids with *Arsenophonus,* compared to those without.

Our detached leaves study allowed us to better understand why Bt3 had higher total nymph deposition counts at the end of the study than Bt1. Similar low performance of Bt1 in comparison to Bt2 and Bt3 was also seen by Chirumamilla et al. (2014). In our experiment we determined that the difference was not due to higher mortality in Bt1, because survival of the two strains was not significantly different, but instead Bt3 starts aphid deposition earlier (Table 1) and deposits more nymphs during peak fecundity (Figure 4). Possible reasons for this difference may be genetic or other factors associated with being in culture longer than Bt3.

Fine scale observation across genotypes on detached leaves also helped us better understand how aphids respond to the different plant genotypes. Our whole plant study (Figure 1) as well as previous research has shown that all three resistant genotypes used in this experiment decrease aphid counts when compared to the susceptible genotype (*W82*) and is further confirmation that *Rag2* impacts Bt1 more than Bt3 (Hill et al., 2010).

Resistant plant genotypes decreased the maximum number of aphids deposited in a single day (Figure 4) as well as delaying the day the first nymph was deposited for both Bt1 and Bt3 strains with the possible exception of Bt3 reared on *Rag2* (Table 1). In addition, *Rag1* and *Rag1 + 2* lowered survival of all the soybean aphid strains used in the experiment (Figure 6).

Previous research has indicated that detached leaves of resistant soybean genotypes depress soybean aphid fecundity (Michel et al., 2010; Lagos-Kutz et al., 2019). Our research is a refinement of previous methods and indicates that detached leaves of the varieties used in this experiment retained levels of host resistance that impacted aphid fecundity in Bt1 and Bt3 aphids. Previous attempts at rearing aphids on detached leaves of biotype differential genotypes used methods that could have accelerated leaf senescence, resulting in decreased ability to mount host resistance modes of action. In our experiment, through direct illumination of plant leaves, frequent replacement of leaves, and the wrapping of the leaf petiole in moistened cotton, we were able to maintain healthy leaves throughout the experiment and the cultures for an extended period (18 days rather than 8 days).

One of the advantages of comparing results from whole plants and detached leaves is that the controlled observation made on detached leaves can compensate for some of the possible factors that negatively impact whole plant cage studies. The patterns observed on whole plants in greenhouses or growth chambers are the result of population dynamics (fecundity, intraspecific competition, survival) and host health and resistance. As was seen in the greenhouse experiments, plant health can be compromised in whole plant experiments as aphid populations increase. Poor host health then reduced aphid populations. This occurs on the hosts most likely to have rapid aphid growth thereby confounding plant health effects with aphid population growth. Individual based models remove this impact by removing intraspecific competition and removing host health effects by maintaining healthy leaves resulting in a closer approximation to the aphid intrinsic rate of increase. Combining individual data with individual based models of population growth therefore isolates the impact of fecundity and host resistance from intraspecific competition and host health. Moreover, the finer observations obtained through individual aphid studies on detached leaves provides fecundity curves necessary for individual based modeling of the evolution of virulence (O’Keefe and Antonovics, 2002; DeAngelis and Mooij, 2005; Schofield et al., 2005).

Previous work has used patterns of fecundity, such as those obtained through this research, in individual based modeling (DeAngelis and Mooij, 2005) to determine optimal refugia with transgenic crops to reduce the evolution of resistance in fall armyworms (Garcia et al., 2016) and pollen beetles (Stratonovitch et al., 2014); to reduce the evolution of pesticide resistance in mosquitoes (Barbosa et al., 2018); and maximize treatments for sea lice on farmed salmon (McEwan et al., 2015). These papers used, as a key parameter of these models, the rate of reproduction of individual organisms. The more detailed information provided by our group combined with other information on aphid movement patterns can result in more precise and better models predicting the evolution of virulence which in turn can be used in Integrated Pest Management (IPM) and resistant plant variety plans. This has been particularly important for increasing the longevity of genetically modified resistant crops including cotton and corn (Ives et al., 2017; Takahashi et al., 2017; Shrestha et al., 2018).

In the detached leaf experiment, all aphid strains had low variability in aphid fecundity across individuals on *W82* but higher variability on resistant genotypes (Figure 7). This is surprising given that the aphid strains used are inbred clonal laboratory strains and that the antibiotic-treated strains are parthenogenetic isofemale lines from which similar responses are expected. We hypothesize that this variable response to plant resistance may result from genetic, epigenetic or symbiont differences that can arise within a clonal line (Figueroa et al., 2018) and/or unequally expressed antibiosis in leaves. Genetic variability within clonal lines has potential for providing an avenue for the evolution of virulence in the soybean aphid. Moreover, this variability in iso-female parthenogenetic laboratory lines, points to the even higher potential for genetic diversity in sexually reproducing and outbred field populations. The high potential for the development of virulence has been observed in field populations of the soybean aphid (Wille and Hartman, 2009). High levels of genic and genotypic diversity have also been observed in the invasive population of *Myzus persicae* in Australia (Wilson et al., 2002). Our observations of range of response of genetic diversity in parthenogenetic clonal laboratory lines of the soybean aphid, as well as previously published observations of the potential for the development of diversity in other clonal aphids (Blackman, 1979; Lushai et al., 2000; Wilson et al., 2002) should be considered when developing a strategy of how to best use resistant plant genotypes in the field for soybean aphid control.

This study shows that symbionts and plant genotypes impact soybean aphid population growth through multiple aspects of fecundity, and when combined result in significant changes in aphid population growth. In addition, host resistance impacts on fecundity that were difficult to observe when analyzing the detached leaf experiment data (Figure 2) were clear when this data was used in individual based population models (Figure 8).

### Future work

The impact of *Arsenophonus* and *Wolbachia* on soybean aphid virulence on resistant soybean genotypes, when aphids and plants are grown in optimal conditions, is difficult to characterize because it is dependent on both the aphid strain and the plant genotype. As both this work and that of others indicate, costs, and benefits due to infections with secondary symbionts can be subtle and difficult to observe fully in the laboratory (Montllor et al., 2002; Koga et al., 2003; Russell and Moran, 2006; Weldon et al., 2013; Oliver et al., 2014; Zytynska and Weisser, 2016). Some of the difficulty arises from the fact that costs and benefits are not only aphid-strain dependent but can also vary among individuals within an aphid strain. It is therefore important that future studies, to detect the impact of facultative symbionts in the soybean aphid, be conducted at a fine scale using individual aphids and we would additionally suggest implementing simulations to identify the effects of host pest interactions on pest population dynamics.

Moreover the effect of *Wolbachia* and *Arsenophonus* in the soybean aphid may be more readily detected when the aphid and its host plant are exposed to stress such as (1) exposure to high and low temperatures, (2) poor plant nutrient status such as low iron, (3) toxin exposure including pesticides, and (4) plant chemistry of the overwintering hosts *Rhamnus cathartica, R. alnifolia* and possibly *Frangula alnus* (syn. *Rhamnus frangula*) (Voegtlin et al., 2004, 2005).

## Data availability statement

The original contributions presented in the study are included in the article/Supplementary material, further inquiries can be directed to the corresponding authors.

## Ethics statement

The manuscript presents research on animals that do not require ethical approval for their study.

## Author contributions

RG, EPW, CBH, and GLH conceived the study. RG, EPW, CBH, RM, AF, AL, AKD, TKH, FNS-A, and MQN carried out the work of setting up experiments, microinjections, testing of aphids infection status, and collected the data. RG, EPW, and CBH analyzed data. EPW wrote the model. RG, CBH, and GLH secured funding. RG and EPW wrote the manuscript. All authors contributed to the article and approved the submitted version.

## Funding

This work was supported by generous grants from the U.S. Mid-West farmers through the checkoff program funds from the United Soybean Board (USB), Illinois Soybean Association (ISA), and the North Central Soybean Research Program (NCSRP) to RG and GLH. Funding was also provided by USDA, Agricultural Research Service, Integrated Management of Soybean Pathogens and Pests, Accession number 432114 to GLH.

## Conflict of interest

The authors declare that the research was conducted in the absence of any commercial or financial relationships that could be construed as a potential conflict of interest.

## Publisher’s note

All claims expressed in this article are solely those of the authors and do not necessarily represent those of their affiliated organizations, or those of the publisher, the editors and the reviewers. Any product that may be evaluated in this article, or claim that may be made by its manufacturer, is not guaranteed or endorsed by the publisher.
