# Supplementary material for: Patterns of asexual reproduction of the soybean aphid, Aphis glycines (Matsumura), with and without the secondary symbionts Wolbachia and Arsenophonus, on susceptible and resistant soybean genotypes
Source: Front Microbiol. 2023 Aug 31;14:1209595. doi: 10.3389/fmicb.2023.1209595 (PMC10501154; doi:10.3389/fmicb.2023.1209595)
Supplement: Supplementary file 1 [file Data_Sheet_1.pdf]

Supplementary Materials

Methods. Antibiotic injections of aphid stocks for the clearance of secondary bacterial symbionts.

The three antibiotic-treated strains Bt1(*A-W-*), Bt3(*A-W-*) and Bt3(*A-W+*) were established using the following methods. The antibiotics ampicillin sodium salt and doxycycline hyclate (Sigma) were used to eliminate *Arsenophonus* and *Wolbachia* bacteria respectively from the parental aphid strains Bt1(*A+W-*), and Bt3(*A+W+*). The antibiotics rifampicin and tetracycline hydrochloride (Sigma) were also tested, but these proved to be toxic for the aphids. Ampicillin and doxycycline were used at concentrations of 50mg/ml and 25mg/ml respectively. Stock antibiotic solutions were prepared in distilled and autoclaved water at a 2X concentration and stored in aliquots at 4°C for up to 2 days. Antibiotics were added in a 1:1 ratio to a 2x injection buffer consisting of 25% glucose and 0.005M PBS (Sigma) in sterile water. The injection buffer was kept frozen in aliquots at -20°C until ready to mix with the antibiotic solution prior to injection.

An injection arena was constructed by filling the bottom of 35x10 mm Falcon plastic petri dishes (Becton Dickinson, Franklin Lakes, NJ) with pink-colored modeling clay (Van Aken, Rancho Cucamonga, CA) to better offset the aphids against the background while being injected. Small convex depressions slightly larger than a soybean aphid were made in the modeling clay around the periphery of the petri dishes using a 5mm diameter dowel. Two- to three-day-old soybean aphid nymphs were placed singly in the convex depressions. Aphids were anesthetized with dry ice by placing the petri dish with the aphids in a covered but not sealed 6x6x3 inches plastic container with ~230 cm^3 of dry ice. Aphids were anesthetized for 8 to 15 minutes and subsequently dehydrated for 3 to 5 minutes in a 6x6x3 inches plastic dehydration chamber containing ~64 cc of anhydrous calcium sulfate ($CaS0_4$) (Drierite, W.A. Hammond, LTD, Xenia, OH) at the bottom of the chamber and covered with dessicant packs and kimwipes. Subsequent to dehydration, aphids were returned to the dry ice chamber until the injection apparatus was ready.

The injection apparatus consisted of a Nanoliter 2000 (Model #B203XVY, World Precision Instruments Inc., Sarasota, FL) attached to a micromanipulator (Brinkmann Eppendorf, Westbury, NY). Microinjection needles were made using 1 mm diameter borosilicate glass capillary tubes with filament (Model #TW100F-4, World Precision Instruments Inc., Sarasota, FL) and pulled using a P97 Flaming /Brown™ micropipette puller with the following settings: Heat= Ramp+5; Pull=90; Velocity=70; in Delay Mode; Delay to 70; Pressure=200units (Sutter Instrument, Novato, CA).

The microinjection needle was filled with mineral oil using a 5ml syringe and mounted on the injector. The needle was trimmed to the desired bore size by cutting it at an angle with a pair of scissors, under 16x magnification. Mineral oil was ejected until 1/5 of the needle was filled from the tip with mineral oil. The needle was then loaded from the tip with as much antibiotic solution that could be accommodated. The micro injector was set to deliver a volume of 7*.5nl* at a slow injection speed. The petri dish with anesthetized

and dehydrated aphids was placed under the microscope and within range of the microinjector. With a fine brush 6 to 8 aphids were positioned on their backs within the convex depressions of the injection arena and the needle positioned to aim for the region between the second and third pairs of legs. Prior to inserting the needle in the aphid a small amount of antibiotic solution was ejected to ascertain that the bore was clear, after which the needle was gently pressed into the abdomen of the aphid until it just pierced the surface of the cuticle. Subsequent to the injection of the prescribed amount of antibiotic solution and visualization of swelling of the aphid abdomen, the needle was held in place for approximately 5 seconds to allow the antibiotic solution to diffuse within the body cavity. While the needle was still inserted in the aphid, a second identical dose of antibiotic solution was delivered in the same manner. The needle was held in place for another five seconds to allow full diffusion. In total 15*nl* of antibiotic solution were injected into each aphid. After completing the injection, the needle was slowly retracted. After each injection the tip of the needle was wiped cleaned with a fine brush dipped in sterile water to help prevent the sugar in the solution from clogging of the needle. The above injection procedure was repeated for all aphids in the petri dish, after which they were allowed to recover for 15 minutes and then were transferred with a fine brush to detached soybean leaves maintained in petri dishes with half cotton rounds imbued with water.

Dishes with injected aphids were inspected the following day. Individuals that survived the injection procedure were placed singly on detached soybean leaves and allowed to reproduce. Offspring from the F1, F2, and F3 generations were injected following the procedure outlined above. The F4 generation was tested for the presence of *Wolbachia* and *Arsenophonus* secondary symbionts using PCR specific primers to *dnaA* or the 16S rRNA gene as listed in the next section below. If an individual was found to be positive the clonal line was discarded; if found negative, offspring were injected again with the respective antibiotic and tested for three subsequent generations. If the offspring of the re-injected clones were found to be consistently negative after the third re-treated generation, the line was maintained without further antibiotic treatment, and checked for infection for the next three subsequent generations when, if found negative, the aphid strain was deemed cured. The bacterial profiles of the aphid stocks used in our experiments are outlined in Table S1. Verification of the aphid stocks can be seen in Fig S1 using the primers in Table S2. All parental and aphid stocks derived from the antibiotic treatments were infected with *Buchnera*. The performance of all lines was compared in the growth chamber, greenhouse and detached leaves experiments.

References

Baldo, L., Bordenstein, S., Wernegreen, J.J. and Werren, J.H. (2006). Widespread Recombination Throughout *Wolbachia* Genomes. Mol. Biol. Evol., 23(2), pp.437–449. doi:https://doi.org/10.1093/molbev/msj049.

Duron, O., Bouchon, D., Boutin, S., Bellamy, L., Zhou, L., Engelstädter, J., et al. (2008). The diversity of reproductive parasites among arthropods: *Wolbachia* do not walk alone. BMC Biol., 6, p.27. doi:https://doi.org/10.1186/1741-7007-6-27.

Everett, K.D.E., Thao, M., Horn, M., Dyszynski, G.E. and Baumann, P. (2005). Novel chlamydiae in whiteflies and scale insects: endosymbionts '*Candidatus* Fritschea bemisiae' strain Falk and '*Candidatus* Fritschea eriococci' strain Elm. Int. Journal of Syst. Evol. Microbiology, 55(Pt 4), pp.1581–1587.

Folmer, O., Black, M., Hoeh, W., Lutz, R. and Vrijenhoek, R. (1994). DNA primers for amplification of mitochondrial cytochrome c oxidase subunit I from diverse metazoan invertebrates. Mol. Mar. Biol. Biotechnol, 3, pp.294–299.

Fukatsu, T., and Nikoh, N. (1998). Two Intracellular Symbiotic Bacteria from the Mulberry Psyllid *Anomoneura mori* (Insecta, Homoptera). Appl. Environ. Microbiol, 64(10), pp.3599–3606. doi:https://doi.org/10.1128/aem.64.10.3599-3606.1998.

Fukatsu, T., Nikoh, N., Kawai, R. and Koga, R. (2000). The Secondary Endosymbiotic Bacterium of the Pea Aphid *Acyrthosiphon pisum* (Insecta: Homoptera). Appl. Environ. Microbiol., 66(7), pp.2748–2758.

Gotoh, T., Noda, H. and Ito, S. (2007). Cardinium symbionts cause cytoplasmic incompatibility in spider mites. Heredity, 98(1), pp.13–20.

Sandström, J.P., Russell, J.A., White, J.P. and Moran, N.A. (2001). Independent origins and horizontal transfer of bacterial symbionts of aphids. Mol. Ecol., 10(1), pp.217–228. doi:https://doi.org/10.1046/j.1365-294x.2001.01189.x.

Tsuchida, T., Koga, R., Meng, X.Y., Matsumoto, T. and Fukatsu, T. (2005). Characterization of a Facultative Endosymbiotic Bacterium of the Pea Aphid *Acyrthosiphon pisum*. Microbial Ecol., 49(1), pp.126–133. doi:https://doi.org/10.1007/s00248-004-0216-2.

Turner, S., Pryer, K.M., Miao, V.P.W., Palmer, J.D. (1999). Investigating deep phylogenetic relationships among cyanobacteria and plastids by small subunit rRNA sequence analysis. Eukaryotic Microbiol., 46(4), pp.327-38. doi: 10.1111/j.1550-7408.1999.tb04612.x

Weeks, A.R., and Breeuwer, J.A.J. (2003) A new bacterium from the cytophaga-flavobacterium-bacteroides phylum that causes sex-ratio distortion. *Insect Symbiosis*. pp.183–194. CRC press. doi: 10:1201/9780203009918.ch11

Zchori-Fein, E., and Brown, J.K. (2002). Diversity of Prokaryotes Associated with *Bemisia tabaci* (Gennadius) (Hemiptera: Aleyrodidae). Ann. Entomol. Soc. Am.,, 95(6), pp.711–718. doi:https://doi.org/10.1603/0013-8746(2002)095[0711:dopawb]2.0.co;2.

Table S1. Profile of *Arsenophonus* and *Wolbachia* bacterial symbionts present in the parental and antibiotic treated *Aphis glycines* strains used in this study. All stocks were infected with *Buchnera*. Parental stocks are indicated with bold font.

Strain	*Arsenophonus*	*Wolbachia*
**Bt1(*A*+*W*-)**	+	-
Bt1(*A*-*W*-)	-	-
**Bt3(*A*+*W*+)**	+	+
Bt3(*A*-*W*-)	-	-
Bt3(*A*-*W*+)	-	+

Table S2. To screen for the presence of *Arsenophonus,* primers were used that amplified the region spanning 16S and the 23S rDNA genes. To screen for *Wolbachia,* primers were used that amplified the *dnaA* gene. To assess the quality of the DNA extraction primers were used that amplified the barcode region of COI.

Organism	Primer Name	Primer Sequence
Arsenophonus[a]	Gly 1-2F	5'-CGCGTMAAGCCAATCTAAGATTG-3'
	480R	5'-CACGGTACTGGTTCACTATCGGTC-3'
Wolbachia[b]	2F	5'-ACAATTGGTTATATCAGCTG -3'
	2R	5'-TACATAGCTATTTGYCTTGG -3'
Positive Control[c]	LCO1460	5'-GGTCAACAAATCATAAAGATATTGG-3'
	HCO2198	5'-TAAACTTCAGGGTGACCAAAAAATCA-3'

[a]Gly 1-2F Primer spanned region between 16S and 23S rDNA genes (This paper)
480 R Primer (Sandström et al 2001).
[b]*dnaA* Primers (Baldo et al. 2006).
[c]barcode cytochrome oxidase I primers (Folmer et al., 1994)

Table S3. Primers used to screen for the presence of symbiotic bacteria in aphid lines used in experiments described in this paper. F = Forward primer; R = Reverse primer

Organism	Expected Size	Primer Name	Primer Sequence	Source
Buchnera 16S	1443bp	RG Buch 16S F	5’-CGAAAGAAAGCTTGCTTTCTTGTC-3’	This paper
		16S 1492R	5’-GGTTACCTTGTTACGACTT-3’	Turner et al., 1999
Spiroplasma 16S	810bp	Spixo-F	5’-TTAGGGGCTCAACCCCTAACC-3’	Duron et al., 2008
		Spixo-R	5’-TCTGGCATTGCCAACTCTC-3’	Duron et al., 2008
Clostridium 16S	500bp	CLO-F	5’-GGAACCTTACCTGGGCTAGAATGTATT-3’	Gotoh et al., 2007
		CLO-R	5’-GCCACTGTCTTCAAGCTCTACCAAC-3’	Gotoh et al., 2007
Hamiltonella 16S	700bp	Hami 92F	5’-TGAGTAAAGTCTGGGAATCTGG-3’	Zchori-Fein and Brown, 2002
		Hami HbR	5’-AGTTCAAGACCGCAACCTC-3’	Zchori-Fein and Brown, 2002
Regiella 16S	200bp	U99F	5’-ATCGGGGAGTAGCTTGCTAC-3’	Sandstrom et al., 2001
		16S B4	5’-CTAGAGATCGTCGCCTAGGTA-3’	Tsuchida et al., 2005
Fritschea 23S	600bp	Fritschea U23 F	5’-GATGCCTTGGCATTGATAGGCGATGAAGGA-3’	Everett et al., 2005
		Fritsch 23SIGR	5’-TGGCTCATCATGCAAAAGGCA-3’	Everett et al., 2005
Cardinium 16S	500bp	CFB-F	5’-GCGGTGTAAAATGAGCGTG-3’	Weeks and Breeuwer, 2003
		CFB-R	5’-ACCTMTTCTTAACTCAAGCCT-3’	Weeks and Breeuwer, 2003
Serratia 16S	480bp	16S A1 (F)	5’-AGAGTTTGATCMTGGCTCAG-3’	Fukatsu and Nikoh, 1998
		16S PASScmp (R)	5’-GCAATGTCTTATTAACACAT-3’	Fukatsu et al., 2000

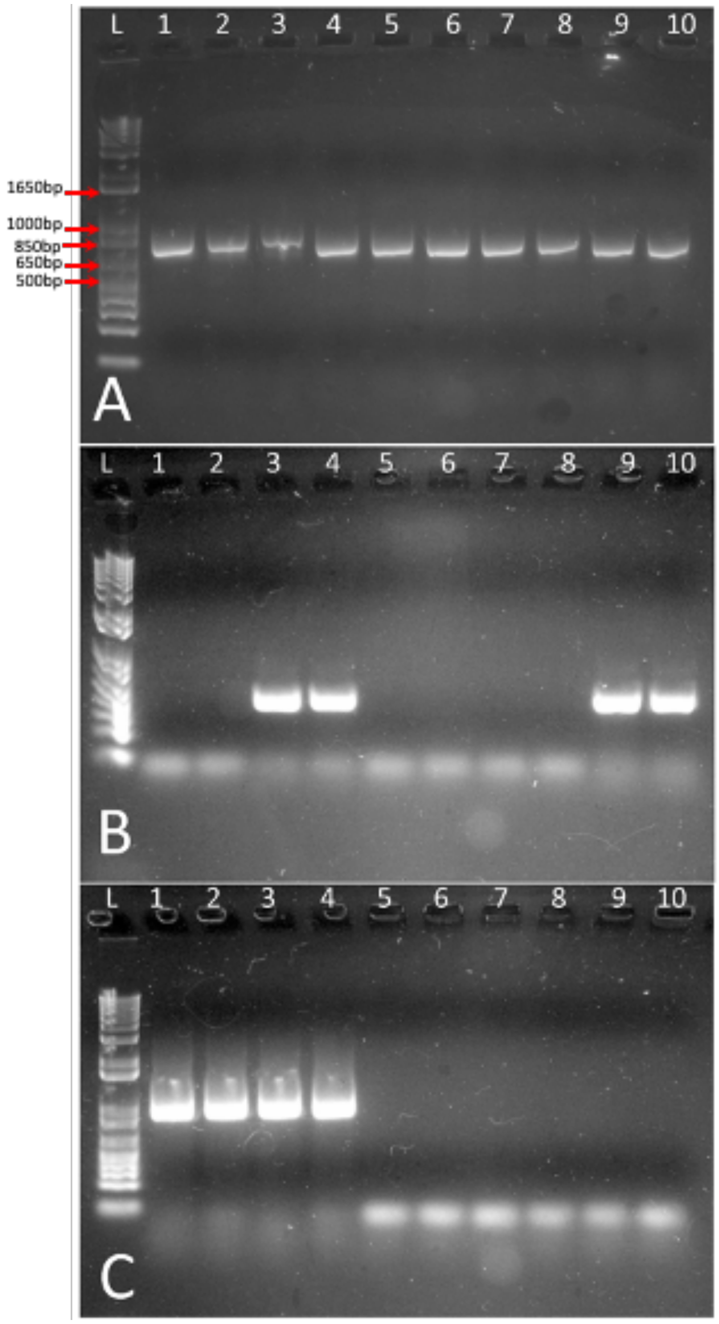

Figure S1. *Arsenophonus* and *Wolbachia* profile of *A. glycines* stocks used in experiments. (A) Positive control for quality of DNA extraction using COI primers LCO 1460 and HCO 2198. (B) Presence of *Wolbachia* checked with primers dnaA 2F and 2R. (C) Presence of *Arsenophonus* checked with primers Gly1-2 F and 480R.Lane designations: L = Ladder; 1,2=Bt1(*A+W-*); 3,4=Bt3(*A+W+*); 5,6=Bt1(*A-W-*); 7,8=Bt3(*A-W-*); 9,10=Bt3(*A-W+*).

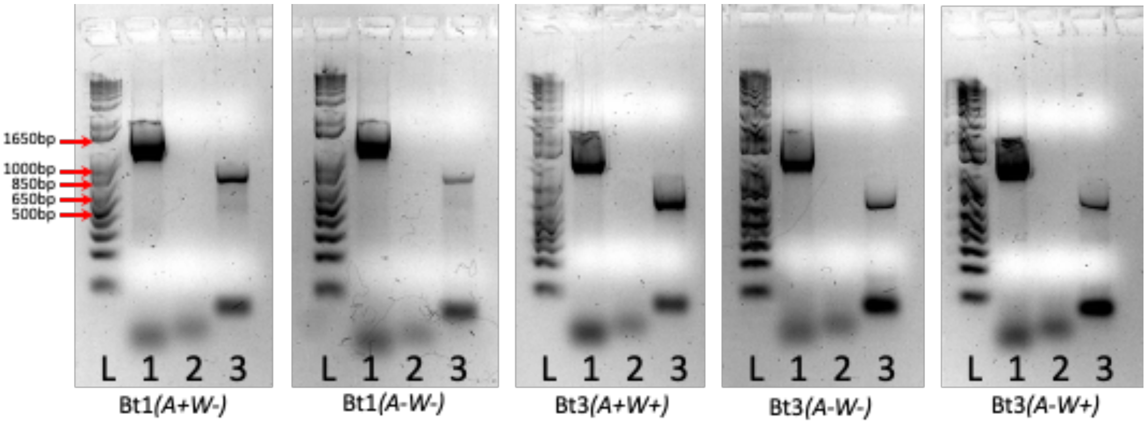

Lane	Endosymbiont	Size	Primer
L	Ladder		
1	*Buchnera*	1443bp	RG Buch 16SF[1] +1492R[2]
2	*Spiroplasma*	810bp	Spixo F[3] + Spixo R[3]
3	*Hamiltonella*	700bp	Hami 92F[4] + Hami R[4]

[1]This paper, [2]Turner et al 1999, [3]Duron et al 2008, [4]Zchori-Fein 2002

Figure S2. Bacterial screen of aphid stocks. PCR reactions using three different bacterial-specific primers to test for the presence of *Buchnera*, *Spiroplasma,* and *Hamiltonella* in the soybean aphid strains used in the experiments. Strains are listed under each gel. Each gel represents one individual. Red arrows indicate key points in the DNA ladder. All strains are infected with *Buchnera* (Lane 1) and *Hamiltonella* (Lane 3), but none are infected with *Spiroplasma* (Lane 2).

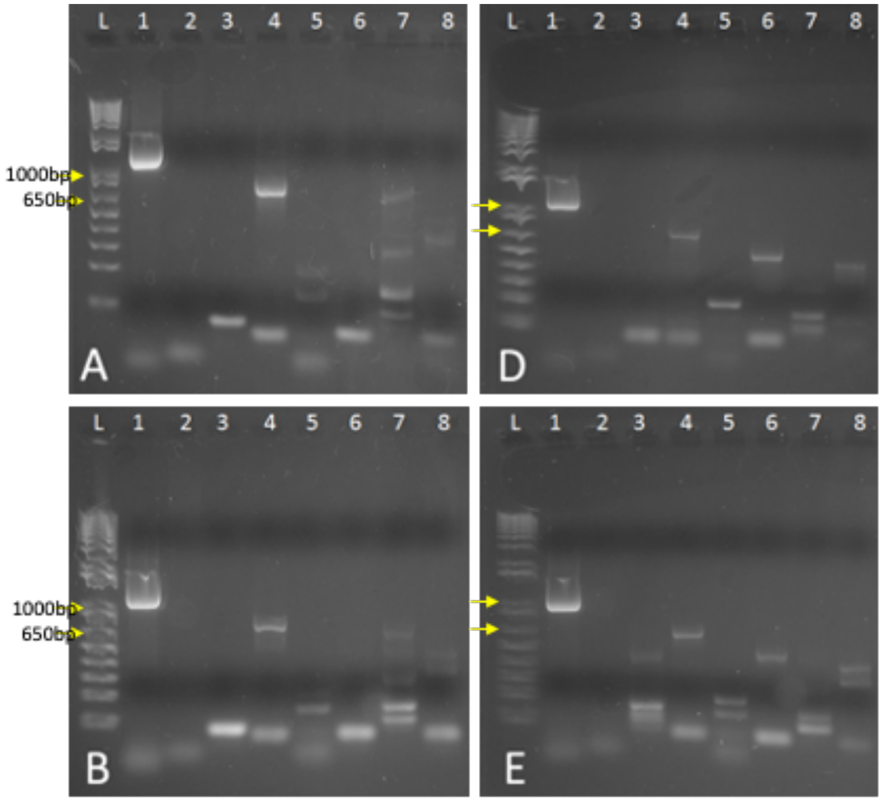

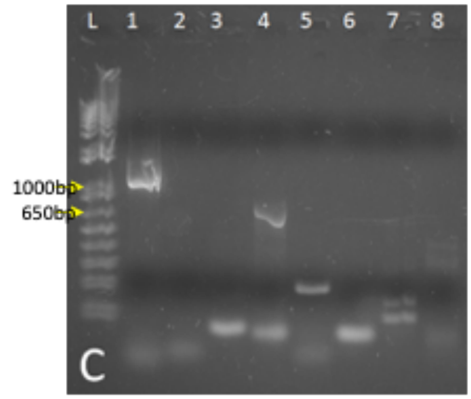

Lane	Endosymbiont	Band	Primer
L	Ladder		
1	*Buchnera*	1443bp	RG Buch 16SF +1492R
2	*Spiroplasma*	810bp	Spixo F + Spixo R
3	*Clostridium*	500bp	Clo F + Clo R
4	*Hamiltonella*	700bp	Hami 92F + Hami R
5	*Regiella*	200bp	Regi U99 F + Regi 16S B4 R
6	*Chlamydia* (*Fritschea*)	600bp	Fritsch U23 F + Fritsch IGR 23S
7	*Cardinium*	500bp	Card CFB F-16S + Card CFB
8	*Serratia*	480bp	Serra PASS + Serra 16S AI

Figure S3. Bacterial screen of aphid stocks. PCR reactions using eight different bacterial specific primers to test for the presence of *Buchnera*, *Spiroplasma*, *Clostridium*, *Hamiltonella*, *Regiella*, *Chlamydia*, *Cardinium* and *Serratia* in the soybean aphid strains: A) Bt1(*A*+*W*-); B) Bt3(*A*+*W*+); C) Bt1(*A*-*W*-); D) Bt3(*A*-*W*-); E) Bt3(*A*-*W*+) used in the experiments. Each gel represents one individual. Lanes and bands are presented in the table. L indicates the 1Kb ladder. Lane 1 *Buchnera* (Positive Control). Expected band size 1443bp, all strains are infected. Lane 4, *Hamiltonella*. Expected band size is 700bp, all strains are infected. All amplifications in lanes 2, 3, 5, 6, 7, 8 are the result of non-specific amplification as they do not have bands of the expected size for the bacteria tested.
